# Asexual Populations of the Human Malaria Parasite, *Plasmodium falciparum*, Use a Two-Step Genomic Strategy to Acquire Accurate, Beneficial DNA Amplifications

**DOI:** 10.1371/journal.ppat.1003375

**Published:** 2013-05-23

**Authors:** Jennifer L. Guler, Daniel L. Freeman, Vida Ahyong, Rapatbhorn Patrapuvich, John White, Ramesh Gujjar, Margaret A. Phillips, Joseph DeRisi, Pradipsinh K. Rathod

**Affiliations:** 1 Departments of Chemistry and Global Health, University of Washington, Seattle, Washington, United States of America; 2 Howard Hughes Medical Institute, University of California San Francisco, San Francisco, California, United States of America; 3 Department of Pharmacology, University of Texas Southwestern Medical Center at Dallas, Dallas, Texas, United States of America; National Institutes of Health, United States of America

## Abstract

Malaria drug resistance contributes to up to a million annual deaths. Judicious deployment of new antimalarials and vaccines could benefit from an understanding of early molecular events that promote the evolution of parasites. Continuous *in vitro* challenge of *Plasmodium falciparum* parasites with a novel dihydroorotate dehydrogenase (DHODH) inhibitor reproducibly selected for resistant parasites. Genome-wide analysis of independently-derived resistant clones revealed a two-step strategy to evolutionary success. Some haploid blood-stage parasites first survive antimalarial pressure through fortuitous DNA duplications that always included the DHODH gene. Independently-selected parasites had different sized amplification units but they were always flanked by distant A/T tracks. Higher level amplification and resistance was attained using a second, more efficient and more accurate, mechanism for head-to-tail expansion of the founder unit. This second homology-based process could faithfully tune DNA copy numbers in either direction, always retaining the unique DNA amplification sequence from the original A/T-mediated duplication for that parasite line. Pseudo-polyploidy at relevant genomic loci sets the stage for gaining additional mutations at the locus of interest. Overall, we reveal a population-based genomic strategy for mutagenesis that operates in human stages of *P. falciparum* to efficiently yield resistance-causing genetic changes at the correct locus in a successful parasite. Importantly, these founding events arise with precision; no other new amplifications are seen in the resistant haploid blood stage parasite. This minimizes the need for meiotic genetic cleansing that can only occur in sexual stage development of the parasite in mosquitoes.

## Author Summary

Malaria parasites kill up to a million people around the world every year. Emergence of resistance to drugs remains a key obstacle against elimination of malaria. In the laboratory, parasites can efficiently acquire resistance to experimental antimalarials by changing DNA at the target locus. This happens efficiently even for an antimalarial that the parasite has never encountered in a clinical setting. In this study, we formally demonstrate how parasites achieve this feat: first, individual parasites in a population of millions randomly amplify large regions of DNA between short sequence repeats of adenines (A) or thymines (T) that are peppered throughout the malaria parasite genome. The rare lucky parasite that amplifies DNA coding for the target of the antimalarial, along with dozens of its neighboring genes, gains an evolutionary advantage and survives. In a second step, to withstand increasing drug pressure and to achieve higher levels of resistance, each parasite line makes additional copies of this region. This second expansion does not rely on the random A/T-based DNA rearrangements but, instead, a more precise amplification mechanism that retains the unique signature of co-amplified genes created earlier in each parasite. Generation of multiple copies of the target genes in the parasite genome may be the beginning of other beneficial changes for the parasite, including the future acquisition of mutations.

## Introduction

The emergence of chloroquine and Fansidar resistance contributed to resurgence of malaria in the 1970s and 1980s [1], [2]. Today, from an estimated 2 billion global clinical cases, ∼0.5 to 1 million individuals die of malaria every year [3], [4], [5]. There is a growing concern that decreased effectiveness of artemisinin combination therapies in Southeast Asia will once again lead to even higher morbidity and mortality [6], [7], [8], [9], [10]. While point mutations and DNA copy number variations have been associated with resistance to previously effective antimalarials [11], [12], [13], [14], [15], a detailed understanding of how haploid blood stages of malaria parasites acquire resistance to truly new antimalarials is critical for the effective management of this global disease.

Similar to what has been observed in clinical settings, *Plasmodium falciparum* malaria parasites are able to acquire resistance under controlled laboratory conditions [16], [17], [18], [19], [20], [21], [22], [23], [24]. Although parasites exposed to potent antimalarials do not show protective, real-time transcriptional responses [25], the targets of novel antimalarials are often definitively revealed in *in vitro* selected resistant parasites through novel mutations or copy number variations in the parasite genome [20], [21], [22], [24], [26], [27], [28]. Such selections are now routinely used to identify target pathways of new antimalarials, but early molecular steps leading to beneficial mutations remain unknown. Here, we use *in vitro* selections to understand how haploid malaria parasite populations, under continual antimalarial pressure, correctly acquire protective changes in their genome. These controlled laboratory selections with asexual blood-stage *P. falciparum* allow step-wise mechanistic dissection of independently evolving parasite cell lines in ways that are not possible in field isolates or other model organisms.

## Results

**Figure 1:**
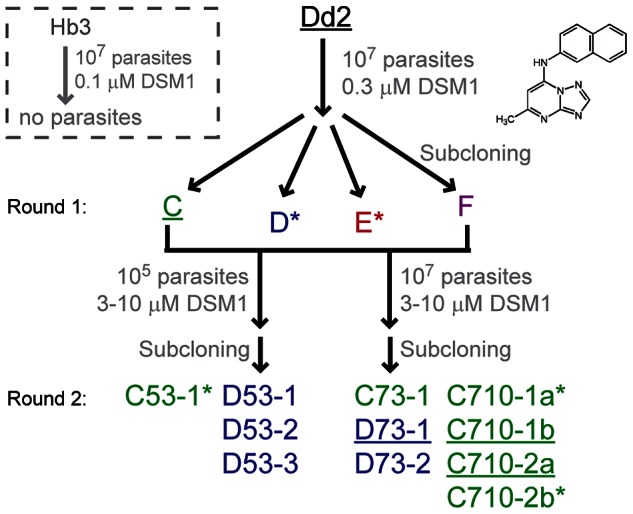
Schematic history of clones selected for varying DSM1 resistance. Color codes are conserved in all figures. Clones used in the drug removal experiments are shown with a “*” and underlined clone names were Illumina sequenced. The round 2 naming convention is as follows: first position, letter of the round 1 clone from which it was derived; second position, number of parasites used in selection (5 refers to 10^5^, 7 refers to 10^7^); third position, concentration of DSM1 used during selection (µM, structure shown as inset on right); last position, refers to clone number.

**Figure 2:**
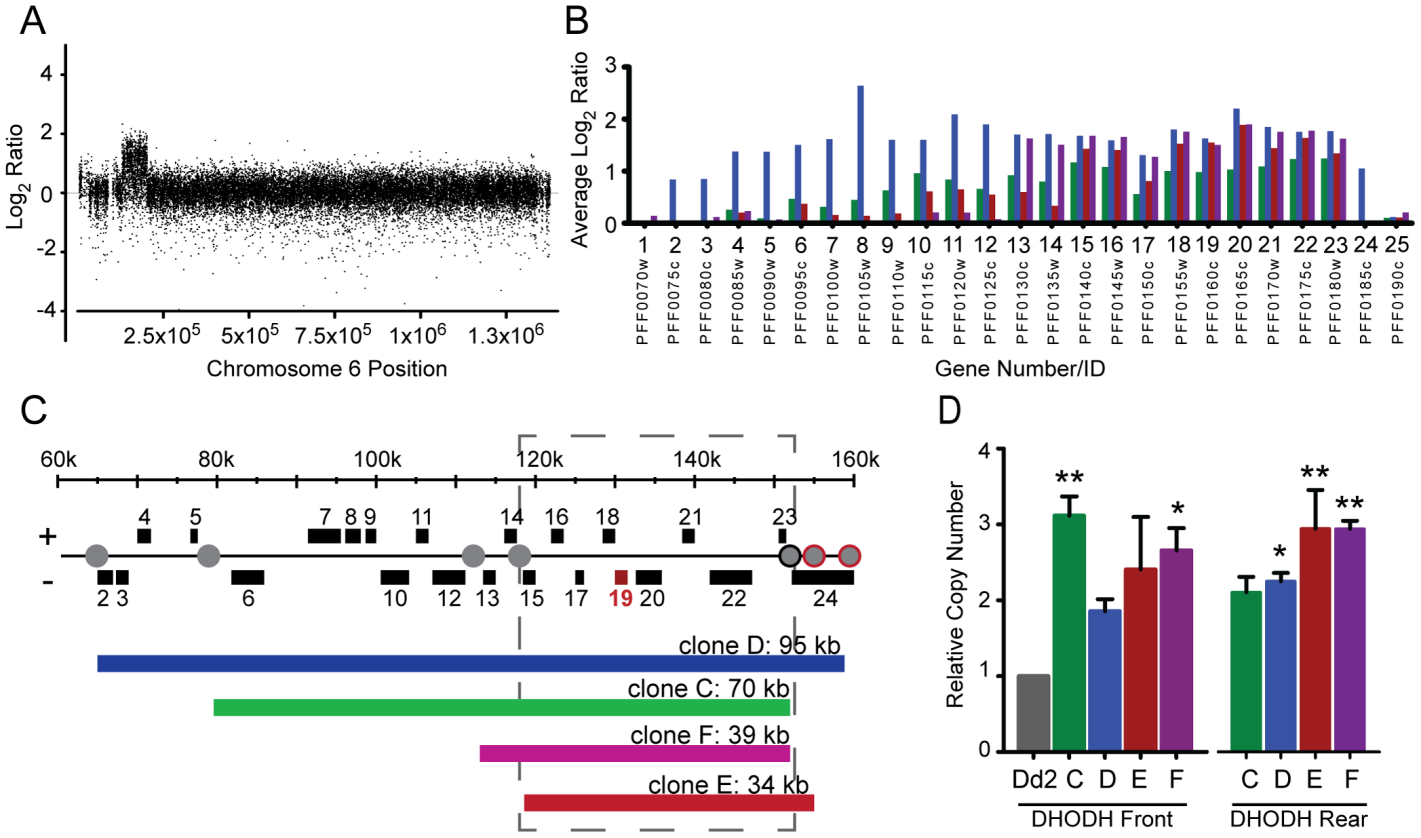
Genes within DHODH amplicons from round 1 clones. **A**. Mid-density microarray results of round 1 clone C (other clones, Fig. S1) showing a 70 kb amplicon at the beginning of chromosome 6 (DHODH amplicon). **B**. Gene coverage (spotted microarray) of the DHODH amplicon (D (blue); C (green); E (red); F (magenta). Average log_2_ ratios are calculated from 3 experimental replicates over 1–3 probes per gene. There were no other significant amplifications detected anywhere in the genome (significance cut off >1.5 fold change, FDR <10%). Gene numbers 1–25 correspond to those listed in Table S7. **C**. Summary of DHODH amplicon size (both spotted and mid-density microarrays). The DHODH target gene (no. 19) is depicted in red, A/T tracks at amplicon junctions are indicated by a grey circle (black outline, shared junction; red outline, junction within introns). The amplicon boundaries of each clone were verified using qPCR (Fig. S8). **D**. Confirmation of DHODH copy number by qPCR. Front and rear primers (Table S12) were used to detect the DHODH gene. Values are relative to Dd2 (grey) and normalized against seryl t-RNA synthetase (PF07_0073) copy number. Error bars depict standard error. Significance was determined against Dd2 (*, p value<0.05 and **, p value<0.005).

**Figure 3:**
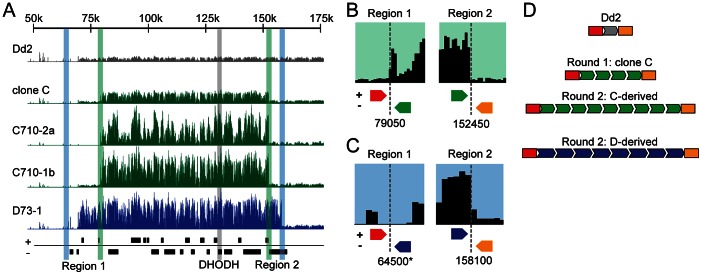
Whole genome sequencing to characterize junctions of DHODH amplicon units. **A**. Histograms of normalized read coverage comparing single-copy Dd2 (grey), round 1, and 2 clones (C-derived, green; D-derived, blue). The scale depicts chromosome 6 position and boxes below represent ORF locations. All histograms are plotted on the same scale; increases in height correlate to increased number of reads from amplifications. Position of DHODH (vertical grey highlight), junction regions 1 and 2 for C (vertical green highlight) and D (vertical blue highlight)-derived clones. **B and C**. Mapping of DHODH amplicon junctions using whole genome sequencing data from C (panel B) and D (panel C)-derived clones. Reads that aligned on either side of the junctions were queried for their paired-end alignments to determine amplicon orientation (Fig. S6). Red arrows; reads that align upstream of the amplicon edge, Yellow arrows; downstream of amplicon edge, Green/blue arrows; within the amplicon. Junction positions for each clone are indicated below histograms. *, reads from this position also map to position ∼1300500 (chromosome 6). **D**. Schematic of the tandem head-to-tail orientation of the various clones and the number of amplified regions (arrows) in the Dd2 parent and each resistant clone.

Resistance was achieved by challenging *P. falciparum* parasites with DSM1, a new potent and selective inhibitor of dihydroorotate dehydrogenase (DHODH) [29] (see structure in inset of Fig. 1). In the initial DSM1 challenge, populations of 10^7^ parasites developed resistance to 0.3 µM DSM1 (Fig. 1, Table S1). Four independently-derived clones, exhibiting ∼5-fold resistance, were selected for further investigation (round 1 clones were designated C, D, E, and F; Table S2). Pair-wise comparative genomic hybridizations of DNA from parent versus DSM1-resistant clones revealed a single ∼2- to 3-fold amplification event on chromosome 6 in all four round 1 clones (Fig. 2A, Fig. S1). The amplicon units ranged in size from 34 to 95 kb, covering 9 to 23 genes (Fig. 2B, C). As discussed below, the variation in the size of the amplicon unit between independently-selected clones provided a molecular fingerprint of each evolving parasite line. All amplicons in each round 1 clone included the DHODH gene (Fig. 2C; gene 19, PlasmoDB gene ID PFF0160c [30], Fig. 2D). DHODH mRNA and protein levels were correspondingly increased (Fig. S2), and mutations were not detected in the gene itself (Fig. S3). Whole genome sequencing of the parent Dd2 clone and clone C (see genome coverage rates in Table S3) confirmed the *de novo* Whole acquisition of the DHODH amplicon and the absence of causal point mutations hidden within individual amplicon units (Fig. 3A; Table S4, Fig. S4). In addition, resistance-associated point mutations were not detected anywhere else in the genome (Table S5, Table 1).

**Table 1:** Summary of SNP verification.

Chromosome	5	5	6	8	14
SNP Position	214184	214244	645035	738807	721985
Gene ID*	PFE0245c	PFE0245c	PFF0750w	MAL8P1_82	PF14_0173
3D7**	T	T	A	T	A
Dd2 (new)	T	T	G	T	T
Dd2 (old)	A	T	G	A	T
C	A	T	G	A	T
D	A	T	G	A	T
E	A	T	Nd	A	T
F	A	T	Nd	A	T
NS	T	T	Nd	T	A
C53-1	A	T	G	Nd	T
D53-1	A	T	Nd	A	T
D73-1	A	T	Nd	Nd	T
D73-2	A	T	Nd	A	T
C710-1a	A	T	G	A	T
C710-2b	A	T	G	A	T

Regions of the genome where non-synonomous exonic SNPs were detected in whole genome Illumina sequencing (Table S5) were PCR amplified (primers listed in Table S12) and dideoxy-sequenced in multiple parasite clones including 3D7 recently acquired from MR4 (MRA-156, MR4, ATCC Manassas Virginia), Dd2 (new) recently acquired from MR4, Dd2 (old) maintained in the Rathod lab for years, round 1 clones (C, D, E and F), NS clone newly selected from Dd2 (new) at 0.3 µM DSM1 (Fig. S10), and round 2 clones (C53-1, D53-1, D73-1, D73-2, C710-1b, C710-2b). Overall, SNPs at positions 214184, 645035, 738807, and 721985 likely originated in the parent Dd2 clone. This conclusion was based on the presence of the wild type nucleotide in the newly selected clone (NS) and/or the mutant nucleotide in an “old” Dd2 clone (the closest known clone to the parent of these selections). The SNP at position 214244 was not detected at all in this analysis, indicating that it was not important for the phenotype. We do not believe that position 214244 is a miscall because we can observe Illumina reads that contain this SNP; the final call was likely due to repetitive sequence in the region that caused misalignment of reads from the proximal SNP position at 214184.

* PlasmoDB gene ID.

** Based on 3D7 sequence from PlasmoDB. Loci on chromosome 6, 8 and 14 were also sequenced from a laboratory 3D7 clone.

Nd, not determined.

To learn how DSM1-resistant parasite populations efficiently arrived at these unique beneficial amplicons, we mapped the junction regions of each independently-derived DSM1 resistant clone. Based on the boundaries initially identified by mid-density microarray analysis (Table S6), we sequenced the DNA between adjoining amplicon units assuming a head-to-tail orientation, and identified long homopolymeric stretches of adenine or thymine (A/T tracks) between the 3′ end of one unit and the 5′ end of the second (Fig. S5). These A/T tracks fall mostly in intergenic regions at the edges of the *P. falciparum* amplicons, with clones C and F sharing exactly the same unit end point (Fig. 2C). The 3′ junction of the remaining two clones D and E exist in two separate introns of PFF0185c (gene 24, Fig. 2C and Table S7). Of the 8 independent events studied here (2 junctions for each of the 4 independently derived clones analyzed), all displayed A/T tracks at the junctions (0 events occurred at a non-A/T tracks). Since homopolymeric tracks of >10 bp make up 5% of the genome [31], the probability that all of the 8 independent events would randomly end with an A/T track is 1 in 25 billion.

Investigations into the orientation of amplicon units (*i.e.* head-to-head or tail-to-tail orientation) as well as whether they were situated outside of chromosome 6 (the original DHODH locus) were expected to provide mechanistic insight into what pathways may be acting at these locations. In a quantitative approach that was not achievable in earlier studies (either by our group (Fig. S5) or others [32], [33]), we acquired paired-end reads from whole genome sequencing that aligned to the junction regions of clone C and D. Histograms of read coverage displayed junctions that were consistent with both microarray and targeted sequencing results discussed above (Fig. 3A). Computationally-isolated reads from the above analysis failed to reveal recombination of the DHODH loci with A/T stretches elsewhere in the genome since reads from all possible junctions aligned to only two genomic locations: (1) the region that represents the reference genome match on chromosome 6 (Fig. 3B, red and yellow arrows) or (2) the opposite end of the amplicon unit (Fig. 3B and C, blue or green arrows; Fig. S6). These data formally prove that the tandem head-to-tail arrangement is the predominant outcome of the initial duplication in DSM1 resistant clones (Fig. 3D).

**Figure 4:**
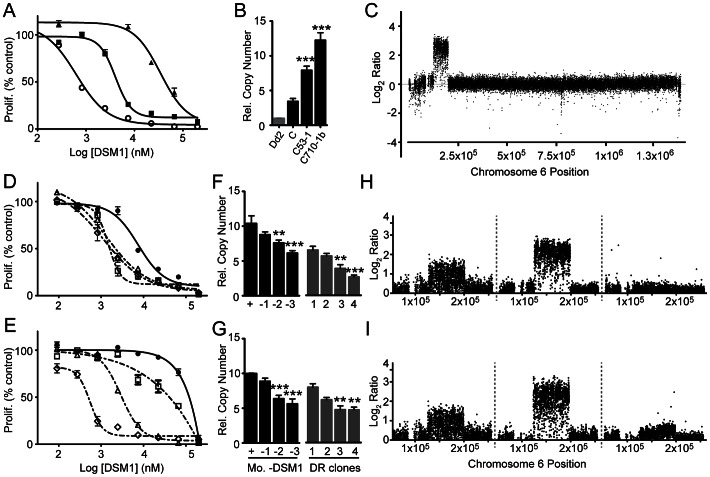
Parallel increases and decreases in DSM1 sensitivities and DHODH amplicons. **A**. Changes in DSM1 sensitivity of Dd2 (open circle) and C-derived clones (C53-1 (square) and C710-1b (triangle)) from a representative dose response experiment (EC_50_ values and full list of experiments, Table S9). **B**. DHODH qPCR showing further amplification in round 2 clones (round 1 C clone included for comparison and used to determine significance). Values are relative to Dd2 (Table S6) and normalized against PF07_0073 and MAL13P1.435. **C**. Mid-density microarray result from a representative round 2 clone C53-1 (relative to Dd2) showing an increased log_2_ ratio of the DHODH amplicon on chromosome 6 (mean log_2_ ratios for all comparisons, Table S6). **D and E**. DSM1dose response curve of C53-1 (**D**) and C710-1a (**E**) populations after growth in the presence (solid line, filled shape) or in the absence (dotted lines, open shapes) of DSM1 for 0 (circle), 1 (square), 2 (triangle), and 3 (diamond) months. **F and G**. DHODH qPCR analysis of C53-1 (**F**) and C710-1a (**G**) populations after 0 to 3 months without DSM1 (black, significance was determined against the +DSM1 population) and −3 month clones (grey, DSM1 removal (DR) clones 1–4). Values are relative to Dd2 and DR clone 1 was used to determine significance. All panels: error bars depict standard error and **, p value<0.005; ***, p value<0.0005. **H and I**. Tuning of the DHODH amplicon: parental amplicon C clone (left, **H** and **I**); round 2 amplicons C53-1 (middle, **H**) and C710-1a (middle, **I**); DSM1 removal amplicons C53-1 DR clone 4 (right, **H**) and C710-1a DR clone 4 (right, **I**). All were compared to Dd2, except DR clones (right, **H** and **I**) were compared to the parental C clone.

Based on outcomes from round 1 clones, we hypothesize that the initial resistance-conferring duplication around the DHODH locus arises from an imprecise, even chaotic, process involving mitotic rearrangement between random A/T tracks that are sprinkled at a high frequency across the genome. Importantly, there appears to be a second non-A/T based step for expanding P. *falciparum* amplicon numbers. When a DSM1 resistant parasite carried more than two units in a freshly-generated amplicon, each unit had the same length, genetic content, and junction regions. Conservation of these units in each independently-selected parasite clone suggested that, after an initial fortuitous duplication between random A/T tracks surrounding the DHODH locus, subsequent expansion of the founder amplicon involves precise homologous recombination that overrides chaotic, possibly unproductive A/T track-based mechanisms. This hypothesis was further tested by exposing round 1 clones to higher DSM1 concentrations (3 µM or 10 µM DSM1 in round 2 compared to 0.3 µM in round 1; Table S8). The resulting independent round 2 clones derived from clones C and D were ∼15- to ∼150-fold more resistant to DSM1 compared to the parent Dd2 (Fig. 4A, Table S9). Comparative genomic hybridizations also showed an increase of the founder DHODH amplicon in these round 2 clones (Fig. 4B and C, Table S6). Whole genome sequencing studies of the amplicon unit junctions of round 2 clones (see genome coverage rates in Table S3) again displayed solely the tandem head-to-tail orientation (Figs. 3B and C and S6). The precise maintenance of the respective founder amplicons in clones C and D is particularly remarkable given that resistance can be conferred by much smaller units as was observed in round 1 clones E and F (Fig. 2C).

To test whether the machinery that allows for faithful expansion of the DHODH amplicons would work with the same precision during deamplification, DSM1 resistant parasites were grown without antimalarial pressure over a long period of time. Overall, resistance of both round 1 and 2 clones initially decreased before stabilizing at ∼2-to 3-fold (Fig. 4D–E, Tables S9 and S10). This observation suggested that there was a measureable fitness cost of maintaining higher levels of the DHODH amplicon, an idea that is consistent with other observations such as the normal growth rate of round 1 clones, the growth defect displayed by round 2 clones (Fig. S7A), and in some cases the increased growth rate following the removal of DSM1 pressure (Fig. S7B).

Similar to what has previously been observed with amplified loci on *P. falciparum* chromosomes 4, 5, and 11 [20], [34], [35], the step-wise decrease of DHODH copy numbers in the absence of DSM1 could be captured over time. Although the starting level of resistance differed, a gradual “dialing down” of the amplicon in the population to stable round 1 levels was observed for two independent C-derived round 2 clones (Fig. 4D–G). Furthermore, comparative genomic hybridization of clones isolated from these cultures grown in the absence of DSM1 for 3 months (DSM1 removal (DR) clones) showed that despite de-amplification, amplicon unit boundaries of C-derived clones were faithfully maintained (Fig. 4H and I, Table S6). Intriguingly, this implied that the pathway that relies on large stretches of homology to “dial up” the amplicon also controls the reverse action and does not allow the A/T track-based mechanism to disrupt amplicon units that were initially evolutionarily successful.

## Discussion

**Figure 5:**
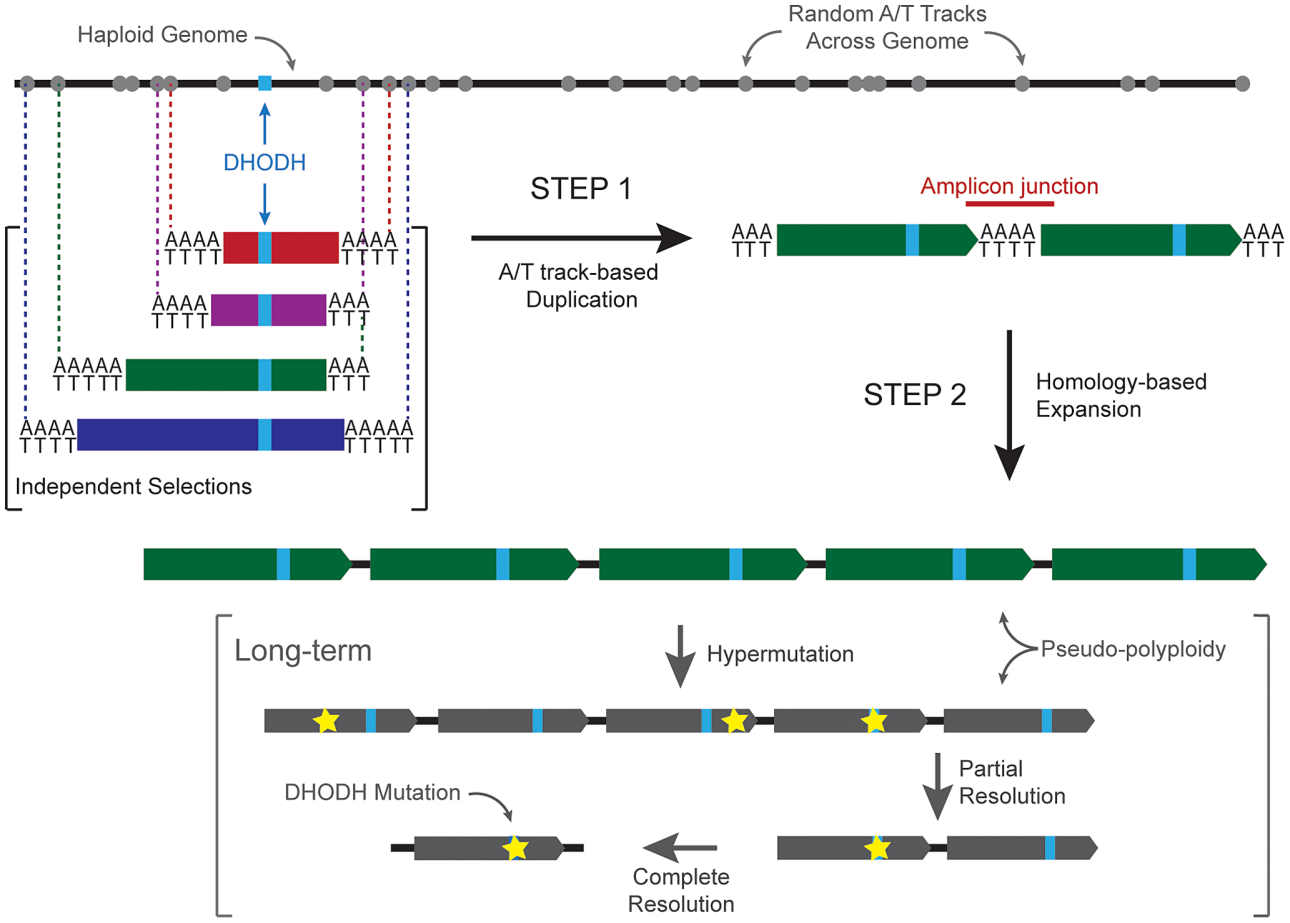
Model of the two-step process that *P.* *falciparum* uses to acquire DNA amplifications. In Step 1, random A/T tracks (grey circles) throughout the haploid genome (black line) initiate a short-homology mediated pathway through presumably either the generation of DNA double-strand breaks due to polymerase pausing or enzymatic action on DNA that is free of histone interactions (see Discussion). In our independent selections, the randomness of the duplication of the genome surrounding DHODH (light blue rectangle) is emphasized by the positions of various initiating A/T tracks (vertical dotted lines) and the generation of differently sized founder amplicons (red, purple, green, and blue bars). The amplicon junction (red line) appears to be generated from uneven “stitching” of the initiating A/T tracks from either side of the amplicon and not simply addition. In Step 2, larger stretches of homology (example green bar) likely trigger homologous recombination-like pathways in the parasite which act to conserve the original beneficial amplicons from Step 1. Long term, the condition of pseudo-plyploidy could allow the generation of mutations (yellow star) across the amplicon, which partial and complete de-amplification could resolve over time.

The DSM1-based selection system offers a precise and reproducible experimental path to understand early events in the evolution of malaria drug resistance, and possibly many other aspects of parasite evolution. Observations of resistance mechanisms against this antimalarial clearly demonstrate that parasites favor *de novo* target amplification to achieve DSM1 resistance and, more generally, that two distinct steps are employed to arrive at beneficial DNA amplifications. In the first step, founder amplicons of independently-selected parasites are established through costly, random duplication of DNA between distant A/T tracks (Fig. 5, Step 1). In the second step, a more precise amplification mechanism efficiently “tunes” the copy numbers of preexisting duplications as needed in response to drug pressure (Fig. 5, Step 2) while avoiding disruption of initial beneficial changes. These detailed insights into how DSM1 resistance is established in malaria parasites raise new important questions regarding the evolution of this organism as a haploid population in a human host. In this environment, parasites encounter host immunity as well as antimalarial drugs, the later often arising in intermittent and changing ways. We believe that the parasite employs unique evolutionary strategies to win these battles without extensive damage in the haploid genome, even before the parasite has a chance to mix with other isolates during the diploid state in the mosquito. These issues are addressed in the subsections below.

### Evolving as a Haploid Genome

The present findings underscore the extraordinary capability of the parasite to evolve during a human infection as a haploid asexual population. In nature, during a single human infection, a few hundred parasites entering the liver expand successfully to become many billions in the face of both drug and immune pressure. Once established in the blood, the parasites can increase and decrease in waves even without a reinfection. In order to evolve during these expansions, haploid parasites must do so with minimal damage to their genome. Similarly to what was first proposed for bacteria [36], the initial random sampling of duplications in the malaria genome under selective pressure serves as an effective first step to locate and identify genetic targets for resistance and generates enough of a foothold for the haploid parasites to proliferate under lethal pressure. The randomness of the initial duplication step in this organism is evident in our detailed molecular characterization of independently-selected resistant parasites from round 1 selections. In addition, these early events also capture the large size of amplicons that are initially sampled (Fig. 2). Assuming one duplicated region of approximately 50–100 kb per parasite, in principle, it is possible to cover the entire 23 Mb *P. falciparum* genome with a few hundred parasites. However, this is clearly not the whole story: the large parasite populations of roughly a million cells required for a successful DSM1 resistance event (Table S1) points to possibly extensive number of “trial duplications” that are non-productive or even lethal to the parasite. The success rates of about 1∶10,000,000 from round 1 selections against this completely novel evolutionary challenge (DSM1) are similar to a previous semi-quantitative estimation of the initial amplification rate in this organism that were inferred from challenges with a traditional aminoquinoline class of antimalarials in clinical use [37].

The few parasites that can identify a productive locus by chance in the first step then rely on a second more efficient step to achieve evolutionarily more robust levels of resistance (Fig. 5, Step 2). Based on survival numbers from round 2 selections (Table S8), this second step appears at least 100-fold more efficient once pseudo-polyploids have been established around a high priority locus. This second process also allows continual fine-tuning of amplicon unit numbers based on the level of antimalarial pressure (Fig. 4). For a haploid blood-stage parasite, when necessary, pseudo-polyploidy could even allow for the safe introduction of point mutations within the amplified region before amplicon units decrease to single copies (Fig. 5, Long Term). Indeed, during laboratory selections, amplifications of the target gene often are observed alongside point mutations in the same gene ([22], [28], [35] and our unpublished observations).

Both during *in vitro* selection and in natural human infection, these productive genomic alterations must take place independent of meiosis. Meiosis is the stage of the life cycle where “textbook” chromosomal crossover mixes different genomes from coinfections to bring together beneficial new traits and to remove damaged DNA in the progeny. However, the sexual stages at which meiosis occurs are not available to the parasite until the transmission of gamete stages to the mosquito. Prior to this stage of the life cycle, how does the evolving haploid parasite avoid large collateral damage as it is under pressure to change in the human? Our detailed characterization of clones from carefully-controlled independent experiments reveals a powerful evolutionary strategy to make precise changes in its genome while expanding in the human, away from the mosquito. At its core, the strategy involves the creation of a single significant new genetic amplification in an individual parasite, even as the entire genome is being sampled by a large starting population. Through controlled laboratory experiments, we directly observed that the amplicon responsible for resistance was the only new amplicon in every individual successful DSM1 resistant parasite. By avoiding adventitious new amplicons elsewhere in the genome, collateral damage is minimized during a time when meiotic cleansings are not available to the parasite. This precise genetic modification is not without cost: every event is accompanied by millions of parasites that do not amplify a useful portion of the genome and do not survive. Whether the initial rearrangements are occurring continuously during the life of the parasites or only in response to stress is a question that remains to be answered.

### Benefits of an AT-rich Genome

The first step in the generation of the DHODH amplicon was clearly mediated by stretches of polyA sequences or polyT sequences (Figs. 5 (Step 1) and S5). Previously, similar homopolymeric A/T tracks have also been identified at the borders of naturally-occurring *P. falciparum* amplicons on chromosome 5 [32], [33], [38], solidifying the relevance of the current laboratory based observations to naturally occurring genomic amplifications in this organism. The A/T-based strategy revealed by these data is uniquely matched with the high AT content of the *P. falciparum* genome, which averages 81% AT but can reach upwards of 90% in introns and intergenic regions [39]. Exactly such approaches are probably not utilized by other *Plasmodium* species that cause human malaria or by other protozoan parasites. Of note, the genomes of the haploid blood stage of *P. vivax*, the second most prevalent human malaria species worldwide [40], averages ∼60% A/T content [41] and Leishmania species that are prone to drug resistance and gene amplifications average ∼40% A/T content [42], [43].

This A/T-dependent approach likely applies to many successful evolutionary selections of different *P. falciparum* parasites; genomic amplifications have been observed during the characterization of both lab-adapted and field-derived parasites from various regions of the world [15], [33], [34], [35], [38]. In the previous studies, however, the exact mechanistic origin of the genomic rearrangements was often ambiguous. First, amplicons were generated in response to antimalarials in clinical use, and independent founder events could not be distinguished from later rearrangements. Second, in some cases, parasites were isolated from clinical infections and thus information on both the clinical drug pressures and the life history of the parasite leading to observed mutational patterns (including passage through a mosquito and recombination with other genotypes) were lost. In the present study, since the DHODH amplicons were selected entirely in the asexual blood stage of *P. falciparum*, we can definitively conclude that the A/T track-mediated step is important in the initial acquisition of a new amplification and not in changing or rearranging amplicons later in evolution.

### Potential Recombination Mechanisms

In addition to showing a general strategy of how a population of parasites narrows in on a resistance-conferring DNA locus, data from the present study points to the importance of two distinct biochemical processes that must operate in each parasite for overall evolutionary success. During replication, A/T tracks are known to cause polymerase pausing due to the rigid bend of the DNA structure [44], [45]. Events that follow could include the creation of a double strand break and recognition by a DNA repair pathway. Alternatively, the rigidness of A/T tracks may prevent adequate histone interactions, leaving DNA open to proteins that may trigger recombination pathways [46]. Recombination pathways generally require large regions of homology to mediate strand invasion but shorter stretches of repetitive bases have also been implicated in the initiation of various mitotic DNA rearrangements [47], [48], [49]. Recent studies of *E. coli* under stress also implicate very short G-rich sequences in template switching between stalled replication forks that leads to the duplication of large genomic regions [50]. In addition to a microhomology-mediated recombination pathway that repairs DNA breaks, a similar replication-based mechanism has been implicated in the generation of complex genomic rearrangements in yeast and humans [49], [51], [52]. Both of these processes appear to get by with extremely small stretches of homology (<10 bp), which are significantly shorter than the A/T tracks observed at the borders of *P. falciparum* amplicons (∼30 bp, Fig. S5) [32], [33], [38]. A/T tracks as large as 60 bp are estimated to make up ∼5% of the parasite genome [31], [39], [53] and although these sequences may take part in template switching or microhomology-mediated recombination as in other organisms, their significant length could also be enough to trigger more canonical recombination pathways requiring as little as 50 bp of homology [54].

### Amplification Verses Point Mutation

Our selection studies with DSM1 show a clear preference for pathways that generate DHODH amplifications even though point mutations have been shown to prevent DSM1 binding to a recombinant catalytically active version of DHODH [55]. Given that round 2 clones display a broad range of DSM1 resistance (Table S9), we had to be sure that hidden point mutations (either in the DHODH gene itself or at other locations in the genome) were not contributing to survival in the presence of high levels of DSM1. Based on the very deep coverage of our whole genome sequencing studies (Table S3, Fig. S4), we are confident that even low frequency mutations within large amplicons would be detected. A few additional observations suggest that resistance is truly due to DHODH amplification and there are no other undetected causal mutations in the DSM1 resistant genome: 1) parasites maintain sensitivity to a number of additional antimalarials (Table S11) indicating that they are not employing a pleotropic resistance mechanism such as drug efflux, and 2) EC_50_ against DSM1 and DHODH copy number decrease in a parallel fashion (Fig. 4), which emphasizes the contribution of the chromosome 6 amplicons to the resistance phenotype (as opposed to changes in other regions of the genome). Despite our confidence in the sequencing data, we cannot rule out that variations in amplicon sizes, and related physiological effects, contribute to the relationship between amplicon copy number and drug resistance.

Why are genome amplifications favored over the acquisition of point mutations in the DSM1 model? The ease with which one can find the correct locus that confers drug resistance and the lack of severe penalties for expanding copy numbers in the neighborhood of the DHODH gene may allow the gene amplification path to dominate. Additionally, the pharmacodynamics of drug exposure during selections could also play a role in favoring amplifications over mutations. Continual, unrelenting drug pressure demands an immediate sustained solution from the parasite population with little tolerance for wrong guesses. Although there is a measurable fitness cost of maintaining many amplicons in the absence of drug pressure (Fig. S7), parasites thrive following the increase in copy numbers of dozens of genes by an order of magnitude. Intuitively, intermittent cycling of increasing antimalarial levels, as is applied in many *in vitro* selection systems, may provide parasites with a chance to acquire mutations that confer high level resistance, and possibly even lose a relevant amplicon that had served its purpose in the early stages of resistance evolution. Beyond this, the nature of the drug, the nature of the target, and its location in the genome could all contribute to the optimum pathway to resistance since continual, uninterrupted application of some antimalarials during laboratory selections (as utilized in our selection scheme) has successfully generated point mutations in various target genes ([19], [23] and additional unpublished work).

### Memory and Generality

The present laboratory-controlled studies show that, in the absence of drug pressure, malaria parasites lose extra copies of amplicons. However, as often seen with field-derived amplicons (Table S13 and [56]), malaria parasites do not always revert back to single copies of the target gene but instead retain a low number of amplicons in the absence of drug pressure (Fig. 4). This act has important implications for future survival: when a parasite population encounters drug pressure that it has successfully overcome before, the population is poised to rapidly re-amplify relevant amplicons quickly and efficiently without heavy collateral damage associated with A/T-based reshuffling between genes near the target.

While the evolution of malaria parasites is studied here in the context of drug resistance as a selection force, the versatile parasite-specific mechanisms that are used to achieve evolutionary success must help the parasites deal with a diverse set of challenges. In the forward direction, acquisition of appropriate beneficial amplifications could help parasites survive antimalarial drugs but also other potential challenges such as host immunity [57]. It may not be a coincidence that the liver stage expansion of an incoming parasite first allows a few hundred parasites to expand to about 100,000 to a million parasites before the population faces unexpected immune-reactions or unusual erythrocyte genotypes of the human patient. In addition to a gain of genetic material through asymmetric recombination, the reverse direction could also have public health relevance. For example, deletions of specific genes in changing parasite populations could render rapid diagnosis tests ineffective [58] thereby misguiding diagnosis-based chemotherapy campaigns.

### Conclusion

The initial two-step evolutionary strategy of *P. falciparum* identified here, likely driven by two different molecular pathways with different biochemical preferences, assists the parasite in finding productive solutions to new and unexpected evolutionary challenges. The strategy is well suited for a parasite population to evolve with minimum collateral damage in surviving cells, it can act to anticipate and mount a rapid response to repeat threats, and it may offer universal advantages to parasite populations that need to withstand multiple threats beyond drug pressure.

## Materials and Methods

### Parasite Culture

For each experiment, erythrocytic stages of *P. falciparum* (previously cloned HB3 or Dd2) were freshly thawed from frozen stocks and maintained as previously described [59], [60], [61]. Briefly, parasites were grown *in vitro* at 37°C in solutions of 2 to 2.5% hematocrit (serotype A positive human erythrocytes) in RPMI 1640 (Invitrogen) medium containing 28 mM NaHCO_3_ and 25 mM HEPES, and supplemented with 20% human type A positive plasma in sterile, sealed flasks, flushed with 5% O_2_, 5% CO_2_, and 90% N_2_. Cultures were maintained with media changes 3 times each week and sub-cultured as necessary to maintain parasitemia below 5%.

### Initial DSM1 Challenge

The highest concentration of DSM1 to which clonal Dd2 and HB3 parasites could develop resistance was determined empirically as previously described [17]. To ensure genetically pure populations, aliquots of 10 infected erythrocytes of each clonal parasite line were allowed to proliferate to about 10^8^ infected erythrocytes. From these populations, 10^2^–10^7^ infected erythrocytes were challenged in flasks with 0.1–10 µM DSM1 (results from 10^7^ are displayed in Table S1). Additionally, 10 infected erythrocytes were challenged with these same concentrations, to ensure that DSM1 was effective and lethal. To confirm that the parasites could proliferate normally under these experimental conditions, one flask of 10 infected erythrocytes did not receive DSM1. This experiment was performed in triplicate, using three independent biological samples of both Dd2 and Hb3 clones. Media was changed 3 times each week (receiving fresh DSM1 each time) and cultures were split 1∶2 once a week to guarantee a continuous supply for fresh erythrocytes during the experiment. Parasite proliferation was monitored by Giemsa-stained thin smear blood samples taken at each media change. Selection flasks were cultured until parasites were observed proliferating or until 90 days, whichever occurred first.

### Selection of DSM1 Resistant Parasites (Rounds 1 and 2)

Using limiting dilution, 10^2^ to 10^7^ (Dd2) or 10^7^ (HB3) populations of genetically pure parasites (see above) were plated across 24 wells of a 96-well plate (each clone was selected in quadruplicate on a single plate). Additionally, a control plate, containing 10 infected erythrocytes per 24 wells, was set up for each clone. To ensure that DSM1 was effective and lethal, the upper half of the control plate was treated with 0.3 µM DSM1. To show that the parasites could proliferate normally under the test conditions, the lower half of the control plate received no DSM1. Plates were cultured (as described above) until parasites were observed proliferating or up to 120 days, whichever occurred first. As soon as parasites were observed (Round 1 results are displayed in Table S2), the well contents were transferred to a new 10 ml culture flask for expansion, sample storage and sub-cloning. During this expansion, DSM1 resistant parasites were kept under continuous 0.3 µM DSM1 pressure and freeze-thawing and culturing for >1month at a time was avoided as much as possible. Four DSM1 resistant clones isolated in round 1 were submitted to another round of selections (round 2). Parasite populations of 10 and 10^7^ were selected with 1, 3.3 and 10 µM DSM1 as described for round 1. In addition, 10^5^ parasites were also challenged with 3.3 µM (Round 2 results are displayed in Table S8). Resistant parasites were isolated as described above before sub-cloning for further analysis.

### Parasite Sub-cloning

To isolate genetically pure populations of DSM1 resistant parasites for further analysis, aliquots of 10–20 infected erythrocytes were plated across an entire 96-well plate. These plates were maintained (as described above) and as soon as parasites were observed proliferating, the well contents were extracted from the plate and transferred to a new 10 ml culture flask for further expansion, sample storage and analysis.

### EC_50_ Determination by Hypoxanthine Uptake Assay

A parasite solution at 0.5–1% parasitemia (0.5% hematocrit) from the clone of interest was plated into a 96-well culture plate. An appropriate range of concentrations of DSM1 (from 0.02–200 µM), depending on the level of resistance of the parasites being tested, were then added to the parasites (because of solubility issues, 100× DSM1 concentrations (in 100% DMSO) were first diluted 1∶10 into RPMI (final 10% DMSO) before being diluted again into the parasite-containing wells (final 1% DMSO)). Each concentration of interest was performed in triplicate and included solvent-only controls. After incubating for ∼48 hours, wells were pulsed with 0.35 µCi each of ^3^H-hypoxanthine. Following an additional 24–40 hours, well contents were extracted and radioactivity was measured. Parasite proliferation in each test well was expressed as a percentage of the solvent control well. EC_50_ values were fit using the GraphPad PRISM software, according to the equation: Y = Bottom+(Top-Bottom)/(1+10^((LogEC50−X) * HillSlope)^).

### Genomic DNA Isolation for Downstream Genomics Methods

For microarrays and quantitative PCR (qPCR) protocols, clonal asynchronous *P. falciparum*-infected erythrocytes were lysed with 0.15% saponin (Akros) for 5 min and genomic DNA (gDNA) was extracted using the DNeasy kit (Qiagen) according to the manufacturer's instructions. For whole genome sequencing, clonal *P. falciparum* cultures (30 mls in T75 flasks, 3% hematocrit) were synchronized with 5% sorbitol for two consecutive cycles (∼45 hrs apart) and then once more (3–4 hr later) before harvesting for gDNA purification. These highly synchronous cultures (∼3% parasitemia at >90% rings) were washed with PBS and frozen at −80°C prior to red blood cell lysis with saponin as above. Isolated parasites were washed 3× with PBS before resuspension in 150 mM NaCl, 10 mM EDTA, and 50 mM Tris-HCl pH 7.5. Parasites were lysed with 0.1% L-loril sarkosil (Teknova) in the presence of 200 µg/ml proteinase K (Fermentas) overnight at 37°C. Nucleic acids were then extracted with phenol/chloroform/isoamyl alcohol (25∶24∶1) pH 7.8–8.1 (Acros) using phase lock tubes (5 Prime). Following RNA digestion (with 100 µg/ml RNAse A (Fermentas) for 1 hr at 37°C), gDNA was extracted twice more as above, once with chloroform, and then ethanol precipitated by standard methods.

### DNA Microarrays and Comparative Genomic Hybridization (CGH)

Spotted DNA microarrays (used for both CGH and expression analysis) consisted of 10,416 −70mer oligonucleotides designed from the *P. falciparum* 3D7 sequence with increased coverage for long ORFs [62]. Additional custom oligonucleotides were included in the microarray to increase coverage of genes involved in folate and nucleic acid metabolism. DNA was spotted on poly-lysine coated slides and post-processed using methods described previously [25], [63]. For hybridizations on spotted DNA microarrays, 5 µg of gDNA from each clone was sheared, labeled with 5-(3-aminoallyl)-2′-deoxyuridine-5′-triphosphate, and coupled to Cy-dyes as was done previously [64]. Uncoupled Cy-dyes were removed using the DNA Clean and Concentrate-5 kit (Zymo Research) before hybridization to the microarray at 62°C for 16–18 h. After washing, slides were dried, scanned at 10 µM resolution using the GenePix 4000B scanner and fluorescent images were quantified with GenePix Pro 3.0 (Axon Instruments). Further analysis, including normalization and statistical methods were performed as described previously [25]. Spotted microarray data are presented in MIAME-compliant format on the NCBI-based Gene Expression Omnibus (GEO) database (Accession # GSE35732).

Commercially manufactured mid-density CGH microarrays containing 385,585 oligonucleotide probes ranging in size from 15- to 45-mer were purchased from NimbleGen Systems, Inc. These microarrays are sufficient to detect copy number variations but not single nucleotide polymorphisms (SNPs) [65], [66]. For hybridizations to mid-density microarrays, gDNA was labeled with Cy3 and Cy5-labeled random nanomers (Trilink Biotechnologies) and hybridized to the current CGH design Plasmodium_3D7_WG_CGH as described previously [65] except hybridization was performed overnight (∼16–18 h) in a 42°C water bath and microarrays were dried and scanned as above (at 5 µM resolution). Normalization and analysis was performed using NimbleScan version 2.6 (SegMNT CGH) and plotted using GraphPad PRISM. Mid-density microarray data are presented in MIAME-compliant format on the GEO database (Accession # GSE37306).

### Quantitative PCR

For DHODH qPCR, two separate sets of primers were used to amplify a 206 bp amplicon beginning at nucleotide +656 of the DHODH coding sequence (DHODH front), and the second set amplified a 158 bp amplicon beginning at nucleotide +1423 of the DHODH coding sequence (DHODH rear) (see Table S11 for all primer sequences). The qPCR protocol was 95°C for 10 min, followed by 39 rounds of 95°C for 15 sec and 60°C for 1 min. For all experiments, we performed melt curves (55°C to 85°C in 0.5°C steps with 1 s hold at each step) to ensure a single amplicon was produced, and standard curves (10× dilution ladders of Dd2 gDNA) to determine the amplification efficiency. Relative copy number was determined for 1 ng of gDNA, using the Pfaffl method [67] according to the equation (E_target_)^ΔCt, target (control−test)^/(E_ref_)^ΔCt, reference (control−test)^, where Seryl t-RNA Synthetase (PF07_0073) and 18 s Ribosomal RNA (MAL13P1.435) served as reference genes. DSM1 resistant clones served as the test, and the Dd2 parent served as the control. Significance was determined from multiple experiments with one-way ANOVA analysis and values from individual clones were compared using the Tukey's Multiple Comparison Test in GraphPad PRISM.

### Whole Genome Sequencing (WGS)

#### I. Library Preparation

Illumina-compatible paired-end libraries were prepared from 50 ng gDNA (see isolation methods described above) using the Nextera DNA Sample Prep Kit (Epicenter Biotechnologies) according to the manufacturer's instructions except that we restricted the bridge PCR step to 6 cycles (instead of 9) and modified the extension step to 65°C for 6 min. Illumina-compatible adapters containing unique barcodes were used at this step instead of Nextera Adaptor 2 so that multiple samples could be run in the same lane of a flow cell by index read sequencing (IDX1 = ‘CGTGAT’: D73-1, IDX2 = ‘ACATCG’: Clone C, IDX3 = ‘GCCTAA’: Dd2, IDX4 = ‘TGGTCA’: C710-1b, IDX5 = ‘CACTGT’:C710-2a). Library fragments from 360 to 540 bp were then size selected on a 5 XT DNA 750 chip using the Lab Chip XT system (Caliper Life Sciences). A final limited-cycle PCR step (Klentaq LA DNA Polymerase (Sigma-Aldrich) with 80% A/T dNTPs) was performed with the outer sequencing adapters (6 cycles of 95°C for 10 sec, 58°C for 30 sec, 60°C for 6 min) in order to enrich for sequence-ready fragments. Prior to cluster generation, library concentrations were confirmed using a high sensitivity DNA Bioanalyzer (Agilent) and qPCR (with Nextera adapter sequences) and samples were pooled at 2 nM in sets of 3. Cluster generation was performed using the cBot HiSeq Cluster Kit v2 (Illumina, Inc.) at a final concentration of 6–8 pM and density of >400 k/mm^2^. Resulting flow cells were run using a v2 HiSeq flow cell on the HiSeq 2000 (Illumina, Inc., ∼90 million reads per lane,Genbank Accession #SRA052245.2).

#### II. Basic analysis

Sequencing reads from individual libraries were separated according to their unique barcodes (introduced during library generation). All reads were aligned to the 3D7 reference genome (PlasmoDB v7.1) using Bowtie [68], allowing a single mismatch for unique reads only. Reads that aligned to multiple regions of the genome were discarded. Genome coverage was estimated as a percentage of the 3D7 genome that was covered by a certain number of reads (see Table S3 for coverage rates). Copy number variations (both amplifications and deletions) in round 1 and 2 clones were identified using histograms of normalized read coverage per million reads aligned over the genome using the Integrated Genome Browser (www.broadinstitute.org/igv). By examining histograms of read coverage across the genome, we detected two deletions evident on chromosomes 2 (position 61539–105810) and 9 (C clone, 1457193–1473789; C710-1b, 1379103–1474063; C710-2a, 1457258–1474460 and D73-1, 1393139–1473966) that were likely due to extended *in vitro* culture [69], [70] and were not considered further. In addition, two amplicons (chromosome 5 (position 888060–970425) and 12 (971307–976534)) that are well described in lab-selected and field-isolated clones (reviewed in [71]) were detected but not considered to contribute to the phenotype because their levels fluctuated between different resistant clones (Table S13).

SNPs were identified by calculating nucleotide frequency for every position in the 5 genomes sequenced independent of the reference genome nucleotide call. These frequencies were used to call the consensus nucleotide and specify amino acid changes if the position is in a coding region. Each clone was independently subjected to this analysis and ultimately compared to the sensitive Dd2 strain to make a ranked list of discordant SNPs. The top 100 SNPs per chromosome were filtered to identify non-synonomous SNPs in exons covered by >5 reads and present in >90% of reads (those from known hypervariable genes, such as pfEMP, rifin, var, and stevor were excluded). These lists were compared between resistant clones and the Dd2 sensitive clone in order to identify SNPs that could be contributing to DSM1 resistance (Table S5). Because the Dd2 sequenced during these studies was not the immediate parent of the DSM1 resistant clones, we verified five SNPs that were present in both round 1 and 2 clones. We performed PCR-directed sequencing (primers listed in Table S11) in multiple parasite clones including 3D7, two Dd2 clones (new, clone acquired from MR4 (MRA-156, MR4, ATCC Manassas Virginia) in June 2011; old, clone used in the lab for several years), all four round 1 clones, and several round 2 clones (Table 1). In addition, these SNPs were investigated in a clone from a recent independent DSM1 selection in which the parent Dd2 clone was known (Dd2 (new) and NS clone 1, Fig. S9). The optimized PCR protocol (95°C for 5 min, 30 rounds of 95°C for 30 sec, 55°C for 1.5 min, and 72°C for 2 min, with a final extension of 72°C for 10 min) gave a single amplified product of the expected size (DHODH-F/R, Table S12). Amplified product was sequenced using an additional 6 internal primers (Seq1–6. Table S12).

#### III. Detecting Mutations in Amplified Regions

To detect SNPs from whole genome sequencing reads in amplified regions, we performed a local BLAST (NCBI, version 2.2.25+) to align reads to nucleotide databases of the amplified region on chromosome 6 with a minimum alignment length of 50 nucleotides and an e-value of <10^−3^. Ungapped alignments were searched for SNPs by calculating nucleotide frequencies per position in the database. SNPs were filtered by percent frequency per nucleotide of the resistant clone over the parent Dd2 and we narrowed our focus on those within open reading frames with a minimum of 20 reads covering any suspected SNP position (presented in Table S4). In addition, Bowtie alignments were converted to the BAM file format for viewing in Integrated Genome Browser and aligned to the 3D7 genome to determine allele frequencies of point mutations across the DHODH gene (presented in Fig. S4).

#### IV. Junction Identification and Orientation of the DHODH Amplicon

Edges of the DHODH amplicon were estimated from WGS read coverage histograms (Fig. 3A). To identify the junctions between amplicons as well as neighboring sequences, we used the paired-end information (matching pairs) for reads at the very edges of the amplicons (Fig. 3B and C). Reads that aligned (using Bowtie [68]) at the red/yellow arrows (+/−200 bp) were isolated and their matching pairs (opposite end of the read in reverse direction, blue/green arrows (Fig. S6A)) were aligned across the entire 3D7 reference genome. The percentage of total reads that align to either side of the amplicon junction were tallied and mapped for each clone (Fig. S6B).

### Accession Numbers

*Plasmodium falciparum* Dihydroorotate dehydrogenase (DHODH), Genbank Accession Number: AB070244.

## Supporting Information

Figure S1 CGH results (mid-density microarrays) for chromosome 6 of round 1 clones. The log_2_ ratio plot for the C clone is displayed in Fig. 2A.
(TIF)

Figure S2 **A**. DHODH mRNA levels for each round 1 clone (C, green; D, red; E, blue; F, magenta) as determined by expression microarrays. Log_2_ ratios from DHODH probes (on spotted DNA microarrays) were converted to relative expression levels and mean values (from 2 separate probes hybridized in triplicate) are plotted with error bars (SEM). One-way ANOVA analysis confirms that the difference between clones is not significant. **B**. DHODH protein levels for each round 1 clone as determined by Western blot analysis. Although only a small region of the blot is shown, no other bands besides that for DHODH (∼65 kD) were visible. A portion of the coomassie stained gel from the same experiment is included as the loading control.
(TIF)

Figure S3 Targeted DHODH sequencing of round 1 and 2 clones. A consensus sequence was generated following assembly of 7 contigs across the 1.7 kb gene for each clone (sequencing primers listed in Table S12) and then compared via ClustalW alignment (Geneious Pro 5.5.6). The green bar displays 100% identity between sequences.
(TIF)

Figure S4 The identification of point mutations across amplified DHODH. Whole genome sequencing reads that aligned to the DHODH gene are scanned for mismatches against the reference 3D7 genome using the Integrated Genome Browser. Histogram bars are colored (green/red) if the allele frequency of a mutant base is >0.05 (1 in 20 reads), otherwise histogram bars are colored in grey. Y axis is presented in a log scale (axis height for each clone is depicted to the right of the plots). Due to the deep coverage of this region of the genome (>50-fold at all nucleotide positions, Table S3), we can confidently conclude that there are no hidden mutations within the amplified DHODH gene. Colored bars in intergenic regions just upstream and downstream of this gene were judged to be sequencing errors based on neighboring repetitive bases.
(TIF)

Figure S5 Summary of results for PCR/sequencing of round 1 amplicon junctions. A. Schematic of approach to PCR across the junction of two amplicons in the same orientation. Primers 1 and 2 vary depending on the clone (primer sequences are listed in Table S12). B. Summary amplicon junctions from each round 1 clone. Presence of the junction is unique to clones with the chromosome 6 amplicon. A1; sequence from the 3′ end of amplicon 1, A2; sequence from the 5′ end on amplicon 2. Sequences were compiled and found to be identical between selected colonies of each round 1 clone (see Text S1) and therefore, only 1 sequence per clone is represented. The starting position (*), A1, and A2 sequence is based on 3D7 genome from PlasmoDB (http://plasmodb.org/plasmo/) (although this data may not exactly match the Dd2 genome, preliminary investigation of Dd2 sequence from the Broad Institute (http://www.broadinstitute.org/annotation/genome/plasmodium_falciparum_spp/MultiHome.html) indicates that this data is reasonably accurate). In all cases (except for the A/T track from clone E (**) which contains 2 T's), “A” followed by a number represents an uninterrupted track adenines of the specified length. For example, “A27” indicates the position of a track of adenines 27 bp long.
(TIF)

Figure S6 WGS-mediated DHODH amplicon junction investigation. **A**. Mapping of DHODH amplicon junctions from WGS paired-end reads. Reads that aligned +/−200 bp surrounding the junctions were queried for their paired-end alignments to the 3D7 reference genome. Panel depicting C clone junction shown for reference (D clone junction, Fig. 3C). **B**. Quantitation of the matching pairs from the initial reads mapping to the windows (A, B, C, D) diagramed in panel A. For Dd2, the matching pair always aligns to the neighboring sequence (A maps to B, B maps to A and C maps to D, D maps to C). For clones containing the chromosome 6 amplicon, the matching pair predominantly aligns to the opposite end of the amplicon (*i.e.* the paired-end reads of region B align to region C and vice versa) indicating a tandem head-to-tail arrangement. Unaligned reads (white box) represent those likely to span the amplicon junction; properties such as low complexity and strain differences limited their alignment to the 3D7 reference genome. *, no initial reads map to this loci due to low genome sequencing coverage. **, alignment is not unique, reads map to another position on chromosome 6 (∼1,300,000). ***, no initial reads map to this loci because of mappability (not unique sequences).
(TIF)

Figure S7 In vitro growth assessment of round 1 and 2 clones in the presence and absence of DSM1. **A**. Growth of DSM1 resistant clones was compared to Dd2 (open circle, solid line) over 6 days in multiple independent experiments. Values from these experiments were combined to determine an overall trend for each set of clones: round 1 (clones C and D) solid square, dashed line; round 2–3 µM resistance (C53-1, D53-3, D73-1) solid diamond, dashed line; round 2–10 µM resistance (C710-1a, 1b, 2a, 2b) solid triangle, dashed line. Percent parasitemia values were normalized to the maximum growth of the Dd2 clone in each experiment and plotted as Mean Normalized Parasitemia. Error bars indicate SEM. Beginning on day 4, there is a statistically significant difference in the parasitemia of round 2 clones compared to Dd2 indicating a growth defect (two-way ANOVA, day by clone interaction F (15,80) = 9.162 and p<0.001, followed by Bonferroni posttests). Round 2–3 uM and −10 uM clones on average grow 54±8 and 50±16% slower compared to wild-type Dd2 clones, respectively. **B**. Growth of C53-1 (left plot, triangle) and C71-1a (right plot, diamond) during DSM1 removal experiments. Parasites were cultured in the presence (closed shape, solid line) or absence (open shape, dotted line) of DSM1 for 45 days before growth was measured as in (**A**) for an additional 14 days and plotted as Mean Normalized Parasitemia (normalization was performed against the maximum growth of the respective –DSM1 clone). Significance could not be determined because only a single value was measured for each time point. While there is no difference in growth between C53-1 ± DSM1, C710-1a (and 2b, data not shown) regains 56% of its growth rate. Growth of Dd2 (grey line) was included for comparison.
(TIF)

Figure S8 qPCR analysis of copy number of various genes across the amplified region of chromosome 6 (italicized genes in Table S7, primers in Table S12). C, green; D, red; E, blue; F, magenta. All values are relative to Dd2 (grey), normalized against seryl t-RNA synthetase copy number (data normalized to the 18 s ribosomal RNA gene displayed similar results), and determined from multiple experiments. Error bars depict standard error. Significance was determined against Dd2 (***, p value<0.0005).
(TIF)

Figure S9 Characteristics of newly selected (NS) DSM1 resistant clones. **A**. EC_50_ plots comparing Dd2 (open circle, EC_50_ value: 0.1±0.01 µM) to uncloned parasites selected with 0.3 µM DSM1 (closed circle, EC_50_ value: 0.3±0.03 µM). Parasite proliferation was measured in triplicate using the hypoxanthine uptake assay and expressed as a percentage of total radioactivity count from the DMSO control. Error bars depict standard error. **B**. qPCR analysis of DHODH copy number in two NS clones (clone 1 mean 5.6±0.4, clone 2 mean 4.8±0.4). Significance was determined relative to Dd2 (***, p value<0.0005).
(TIF)

Table S1 Summary of initial DSM1 challenge results. Dd2 (DSM1 EC_50_ of 0.2 µM) and HB3 (DSM1 EC_50_ of 0.06 µM) infected erythrocytes were challenged with various concentrations of DSM1 to determine the highest level of achievable resistance. Population sizes of 10^2^–10^6^ Dd2 or Hb3 parasites were also tested but not able to develop resistance to 0.1 µM or higher concentrations of DSM1 (unpublished data).
(DOC)

Table S2 Round 1 selections. 10^7^ Dd2 parasites were plated over 24 wells (4 replicates) and challenged with 0.3 µM DSM1 (Note: populations of <10^7^ parasites were not able to survive treatment with this DSM1 concentration). In total, 8 wells were positive for resistant parasites (round 1 clones). Four of these wells were randomly selected for DSM1 EC_50_ determination and sub-cloning. One sub-clone of each round 1 clone was selected for further analysis. Nd, EC_50_ not determined.
(DOC)

Table S3 Whole genome sequencing coverage rates of various regions of interest across the *P. falciparum* genome. Overall very deep coverage was achieved in all clones except C710-1b clone (designated “*”). Whole genome rates are considerably lower than those for the DHODH gene presumably due to the inclusion of intergenic regions in this data set where base composition may limit the unique alignment of many reads. Coverage rates within clone C and D amplicon boundaries are included to emphasize the very deep coverage of these areas and thus, our confidence in the lack of mutations across these regions. Nd, not determined.
(DOC)

Table S4 Summary of mutations in the DHODH amplicon. In order to find low frequency mutations in an amplified region, positional nucleotide frequencies were identified by comparing Illumina reads from resistant clones and Dd2 that cover the amplicons on chromosome 6. This result of this analysis across the DHODH gene is also summarized in Fig. S4. DHODH (PFF0160c) is the target of DSM1. (−) no SNPs detected.
(DOC)

Table S5 SNPs present in >90% of Illumina sequencing reads. The top 5 listed SNPs were validated by PCR and sequencing (Table 1) and shown to preexist in Dd2 (see Materials and Methods section of main paper).
(DOC)

Table S6 Summary of chromosome 6 amplicon boundaries and DHODH copy numbers for key round 1 and 2 clones. Microarray probe positions were judged from mid-density microarray analysis as the first (Start) and last (Stop) probe that exhibited a log_2_ ratio >0.3 in the amplicon border region. Mean log_2_ ratios were calculated across the entire amplified region. Since exact DHODH copy numbers could not be estimated from amplicons exhibiting log_2_ ratio >0.8, qPCR was employed (mean of values for front DHODH primer set (Table S12) from multiple experiments). Clones from two DSM1 removal (DR) experiments in which CGH analysis was performed are listed underneath the clone in which they were derived. All CGH experiments are pair-wise comparisons against Dd2 genomic DNA (except DR clones are compared to the round 1 C clone). Nd, not determined.
(DOC)

Table S7 Description of genes contained within the largest DHODH amplicon. The DHODH target of DSM1 is bolded. Other genes selected for qPCR analysis (see Table S12) are italicized. The smallest amplicon (from clone E) encompasses gene numbers 15 to 23.
(DOC)

Table S8 Round 2 selections. Populations of parasites were challenged with 1–10 µM DSM1 and scored for positive growth over 48 (for an initial population of 10^1^) or 96 days (all other conditions). Resistant parasites from parental clone C and D were sub-cloned for further analysis (*) but those from clones E and F were not followed further. Nd, not determined.
(DOC)

Table S9 Summary of EC_50_ values for round 2 clones. Values were determined using a higher range of DSM1 (0.09–200 µM) than was used for round 1 clones. These concentrations approach saturation for this compound, which may explain the increase in Dd2 EC_50_ (compared to value in Table S2) and high variability observed in these experiments. Fold increase was calculated against Dd2 from this table (tested at the high range of DSM1). Results from drug removal (DR) experiments are listed underneath the clone in which they were derived from (−1: 1 month without DSM1). Nd, could not be determined.
(DOC)

Table S10 EC_50_ values for DSM1 resistant round 1 clones D and E after 8 months of continuous culture with or without (*) 0.3 µM DSM1 pressure. Fold-resistance values are included in parentheses for ease of comparison.
(DOC)

Table S11 Additional antimalarial EC_50_ determination (±95% CI) for DSM1 resistant clones. Proguanil and 1843U89 target the *P. falciparum* dihydrofolate reductase enzyme and thymidylate synthase, respectively [72]. 5-Fluoroorotate inhibits pyrimidine biosynthesis in *P. falciparum* [73], [74]. Artemisinin is currently used clinically and its target remains unidentified (reviewed in [75]). Assay type A is a flow cytometry-based method that involves measurement of parasitemia using SYBR green and assay type B depends on the uptake of radiolabeled hypoxanthine (see Materials and Methods and Text S1). (−), experiment not performed. Nd, could not be determined.
(DOC)

Table S12 Summary of primers by experiment.
(DOC)

Table S13 Copy number assessment of the chromosome 5 and 12 amplicons in DSM1 resistant clones using microarray and qPCR.
(DOC)

Text S1 Supplementary Materials and Methods. This document contains the following sections: Expression Analysis, Determination of DHODH Protein Levels, Targeted DHODH Sequencing, EC_50_ Determination by SYBR Green Assay, PCR and Sequencing of the DHODH Amplicon Junction, Growth Assessment, Quantitative PCR of other genes contained within the DHODH amplicon, Abbreviations, and Supplementary References.
(DOCX)
