# Supplementary figures and images for: Asexual Populations of the Human Malaria Parasite, Plasmodium falciparum, Use a Two-Step Genomic Strategy to Acquire Accurate, Beneficial DNA Amplifications
Source: PLoS Pathog. 2013 May 23;9(5):e1003375. doi: 10.1371/journal.ppat.1003375 (PMC3662640; doi:10.1371/journal.ppat.1003375)

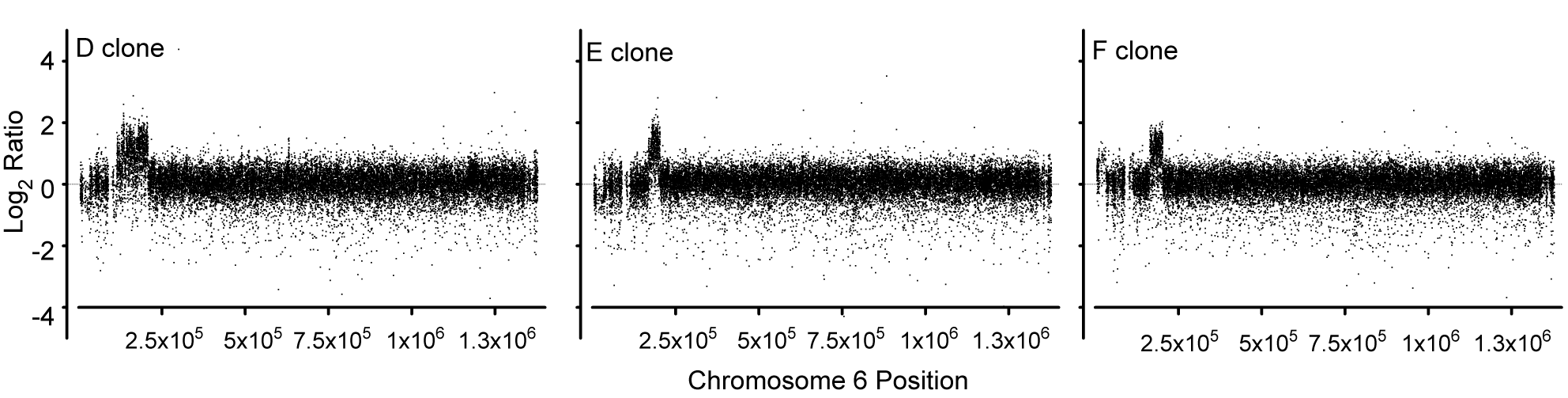

Supplement: Figure S1 — CGH results (mid-density microarrays) for chromosome 6 of round 1 clones. The log2 ratio plot for the C clone is displayed in Fig. 2A. (TIF) [file ppat.1003375.s001.tif]

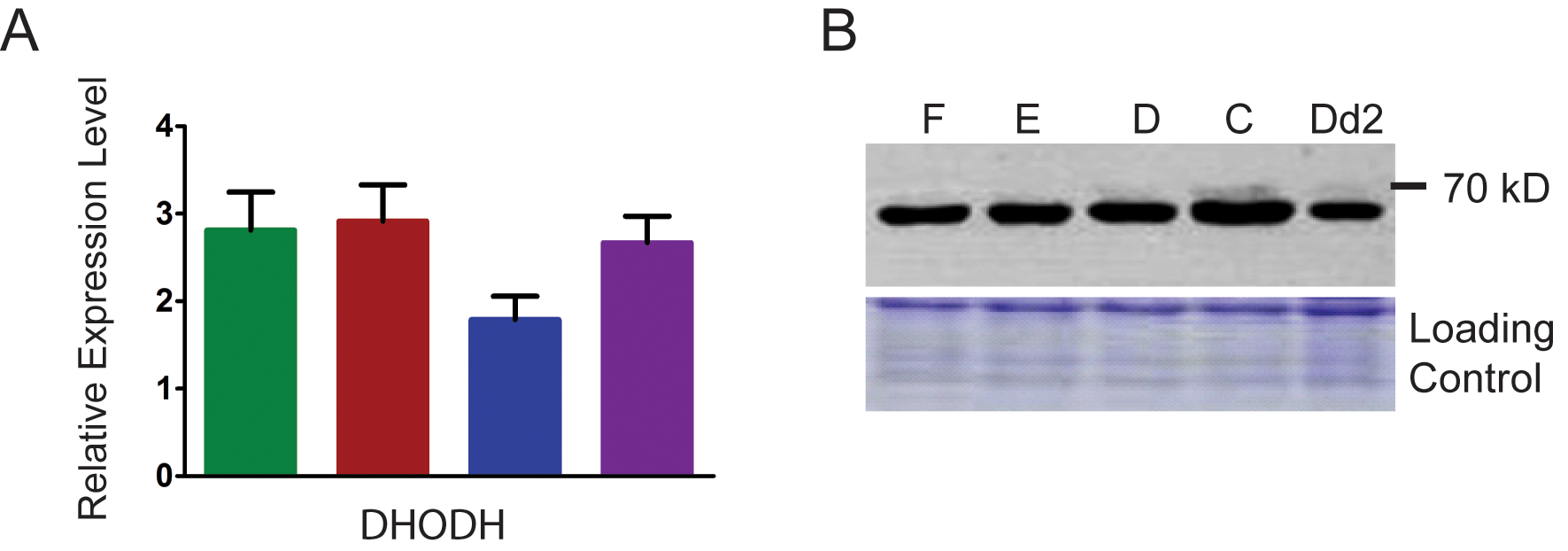

Supplement: Figure S2 — A. DHODH mRNA levels for each round 1 clone (C, green; D, red; E, blue; F, magenta) as determined by expression microarrays. Log2 ratios from DHODH probes (on spotted DNA microarrays) were converted to relative expression levels and mean values (from 2 separate probes hybridized in triplicate) are plotted with error bars (SEM). One-way ANOVA analysis confirms that the difference between clones is not significant. B. DHODH protein levels for each round 1 clone as determined by Western blot analysis. Although only a small region of the blot is shown, no other bands besides that for DHODH (∼65 kD) were visible. A portion of the coomassie stained gel from the same experiment is included as the loading control. (TIF) [file ppat.1003375.s002.tif]

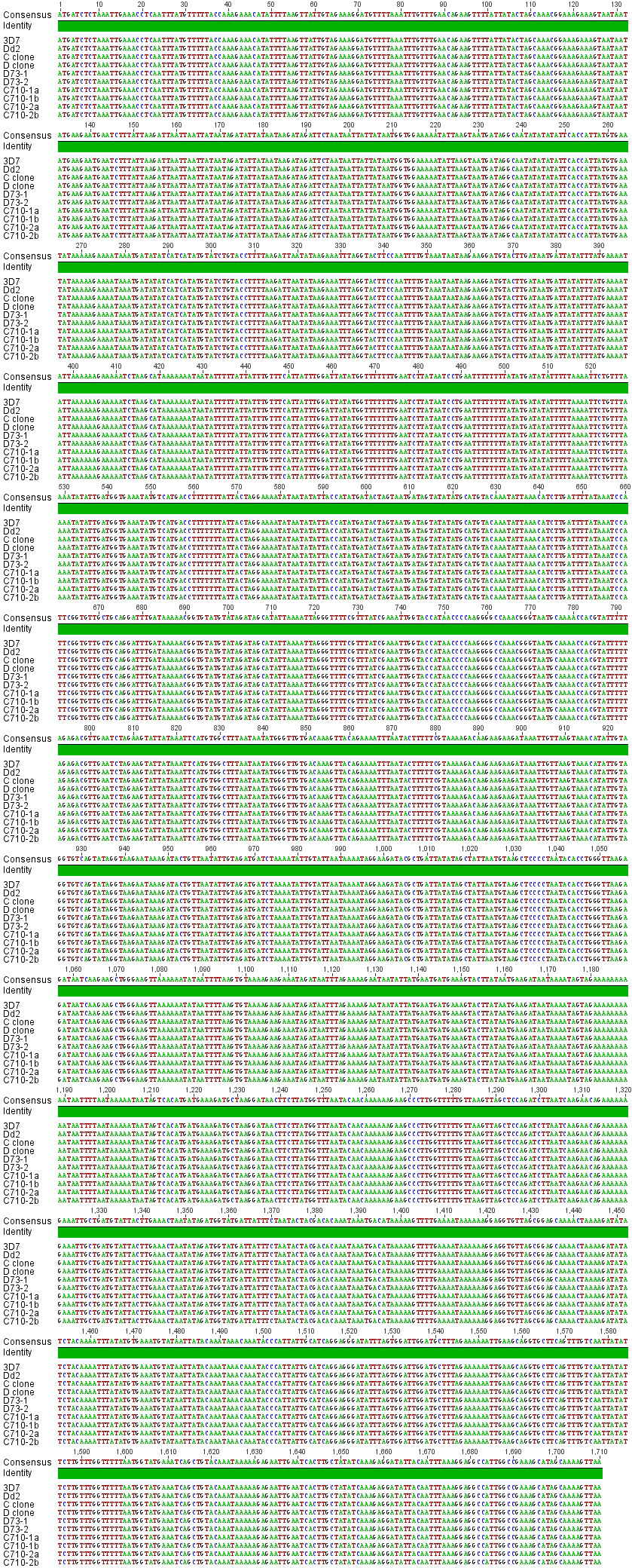

Supplement: Figure S3 — Targeted DHODH sequencing of round 1 and 2 clones. A consensus sequence was generated following assembly of 7 contigs across the 1.7 kb gene for each clone (sequencing primers listed in Table S12) and then compared via ClustalW alignment (Geneious Pro 5.5.6). The green bar displays 100% identity between sequences. (TIF) [file ppat.1003375.s003.tif]

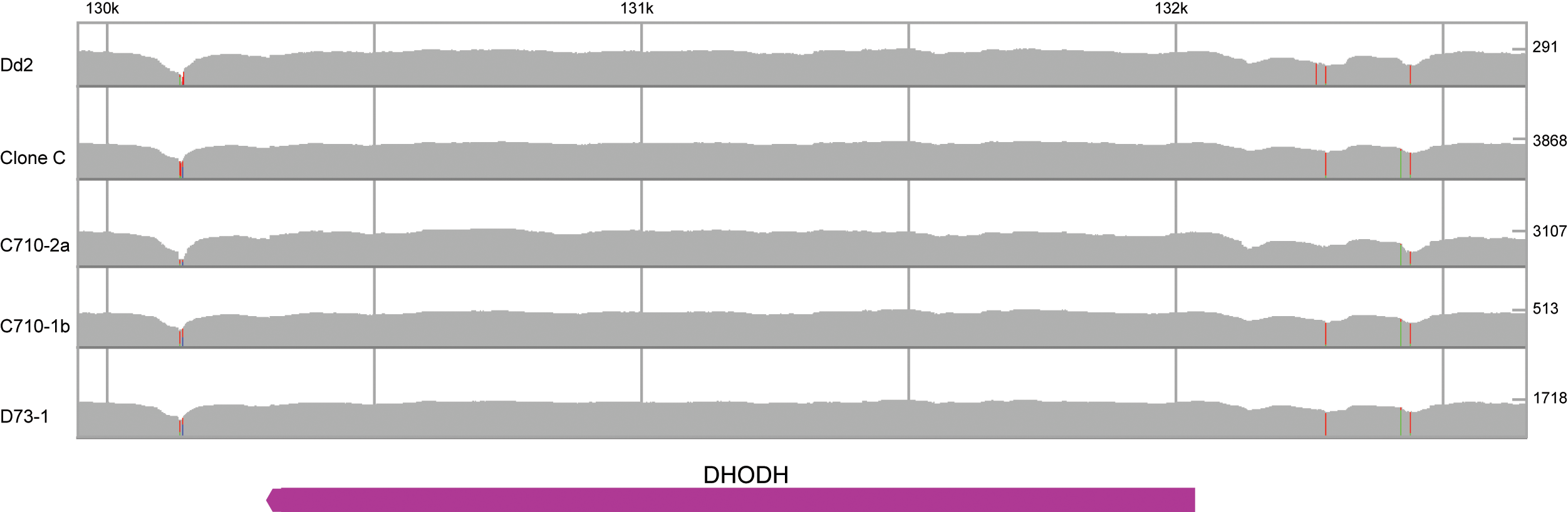

Supplement: Figure S4 — The identification of point mutations across amplified DHODH. Whole genome sequencing reads that aligned to the DHODH gene are scanned for mismatches against the reference 3D7 genome using the Integrated Genome Browser. Histogram bars are colored (green/red) if the allele frequency of a mutant base is >0.05 (1 in 20 reads), otherwise histogram bars are colored in grey. Y axis is presented in a log scale (axis height for each clone is depicted to the right of the plots). Due to the deep coverage of this region of the genome (>50-fold at all nucleotide positions, Table S3), we can confidently conclude that there are no hidden mutations within the amplified DHODH gene. Colored bars in intergenic regions just upstream and downstream of this gene were judged to be sequencing errors based on neighboring repetitive bases. (TIF) [file ppat.1003375.s004.tif]

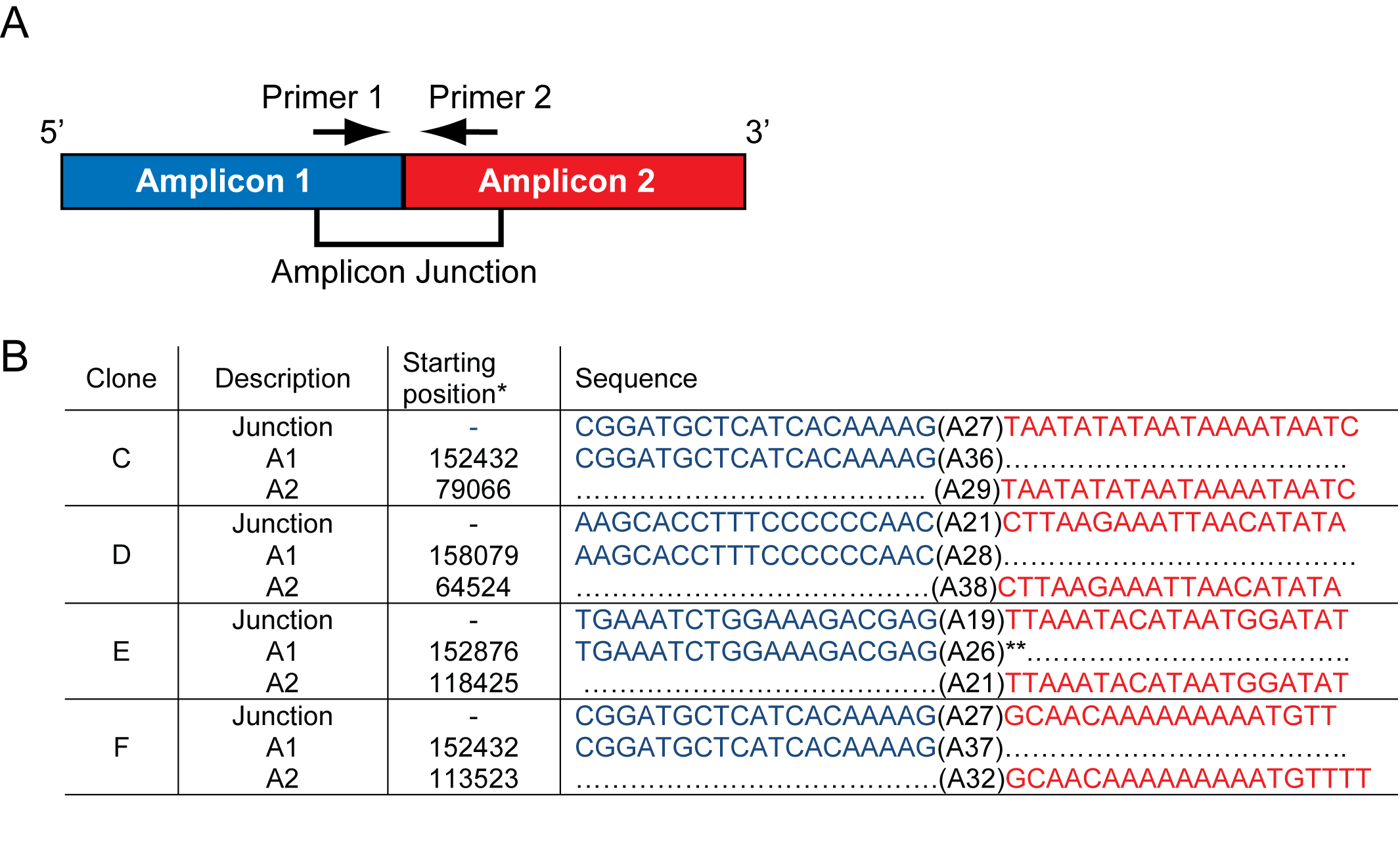

Supplement: Figure S5 — Summary of results for PCR/sequencing of round 1 amplicon junctions. A. Schematic of approach to PCR across the junction of two amplicons in the same orientation. Primers 1 and 2 vary depending on the clone (primer sequences are listed in Table S12). B. Summary amplicon junctions from each round 1 clone. Presence of the junction is unique to clones with the chromosome 6 amplicon. A1; sequence from the 3′ end of amplicon 1, A2; sequence from the 5′ end on amplicon 2. Sequences were compiled and found to be identical between selected colonies of each round 1 clone (see Text S1) and therefore, only 1 sequence per clone is represented. The starting position (*), A1, and A2 sequence is based on 3D7 genome from PlasmoDB (http://plasmodb.org/plasmo/) (although this data may not exactly match the Dd2 genome, preliminary investigation of Dd2 sequence from the Broad Institute (http://www.broadinstitute.org/annotation/genome/plasmodium_falciparum_spp/MultiHome.html) indicates that this data is reasonably accurate). In all cases (except for the A/T track from clone E (**) which contains 2 T's), “A” followed by a number represents an uninterrupted track adenines of the specified length. For example, “A27” indicates the position of a track of adenines 27 bp long. (TIF) [file ppat.1003375.s005.tif]

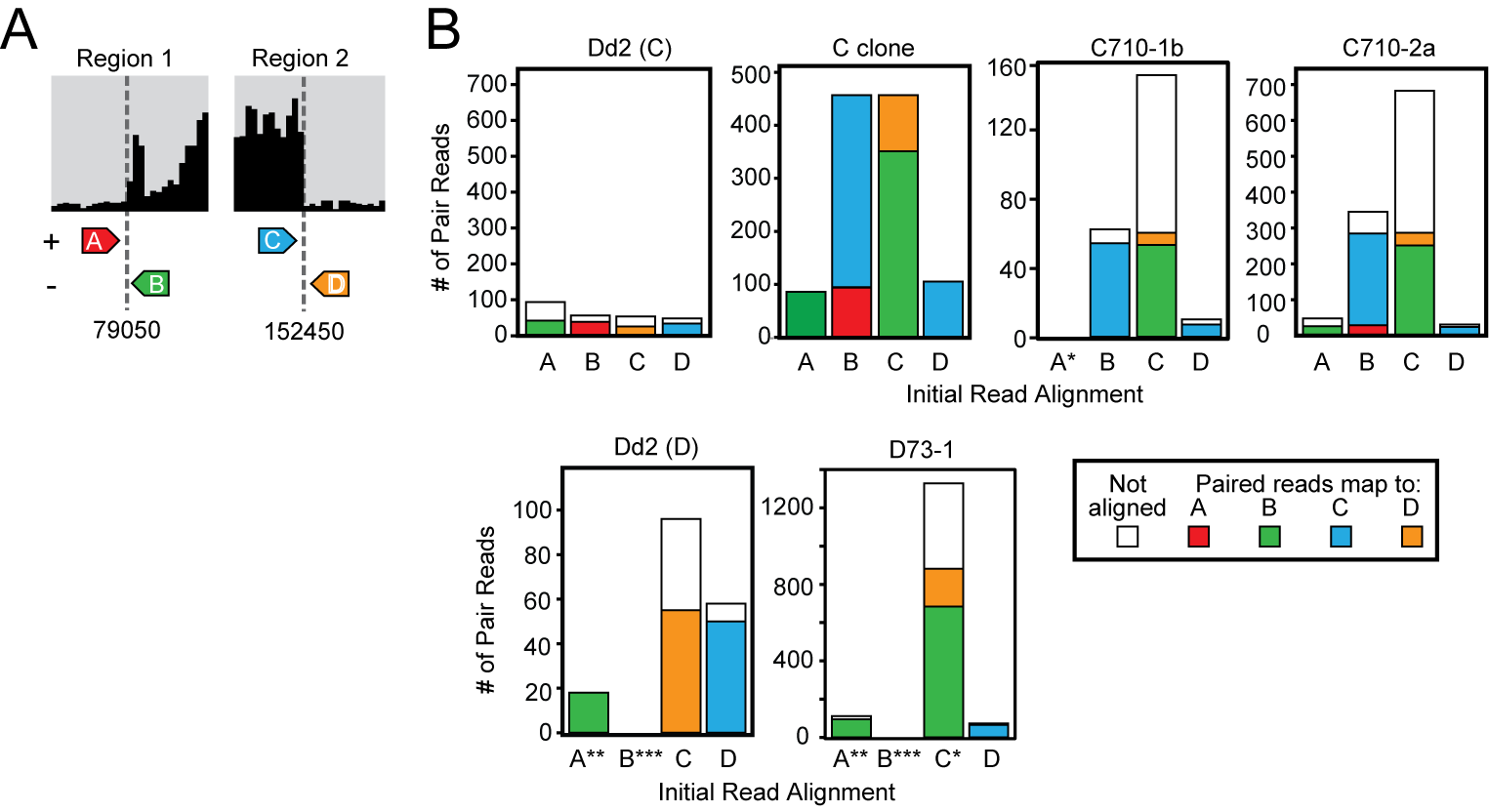

Supplement: Figure S6 — WGS-mediated DHODH amplicon junction investigation. A. Mapping of DHODH amplicon junctions from WGS paired-end reads. Reads that aligned +/−200 bp surrounding the junctions were queried for their paired-end alignments to the 3D7 reference genome. Panel depicting C clone junction shown for reference (D clone junction, Fig. 3C). B. Quantitation of the matching pairs from the initial reads mapping to the windows (A, B, C, D) diagramed in panel A. For Dd2, the matching pair always aligns to the neighboring sequence (A maps to B, B maps to A and C maps to D, D maps to C). For clones containing the chromosome 6 amplicon, the matching pair predominantly aligns to the opposite end of the amplicon (i.e. the paired-end reads of region B align to region C and vice versa) indicating a tandem head-to-tail arrangement. Unaligned reads (white box) represent those likely to span the amplicon junction; properties such as low complexity and strain differences limited their alignment to the 3D7 reference genome. *, no initial reads map to this loci due to low genome sequencing coverage. **, alignment is not unique, reads map to another position on chromosome 6 (∼1,300,000). ***, no initial reads map to this loci because of mappability (not unique sequences). (TIF) [file ppat.1003375.s006.tif]

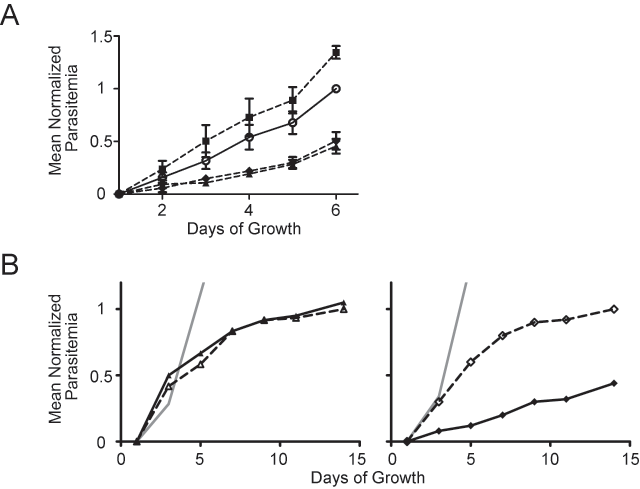

Supplement: Figure S7 — In vitro growth assessment of round 1 and 2 clones in the presence and absence of DSM1. A. Growth of DSM1 resistant clones was compared to Dd2 (open circle, solid line) over 6 days in multiple independent experiments. Values from these experiments were combined to determine an overall trend for each set of clones: round 1 (clones C and D) solid square, dashed line; round 2–3 µM resistance (C53-1, D53-3, D73-1) solid diamond, dashed line; round 2–10 µM resistance (C710-1a, 1b, 2a, 2b) solid triangle, dashed line. Percent parasitemia values were normalized to the maximum growth of the Dd2 clone in each experiment and plotted as Mean Normalized Parasitemia. Error bars indicate SEM. Beginning on day 4, there is a statistically significant difference in the parasitemia of round 2 clones compared to Dd2 indicating a growth defect (two-way ANOVA, day by clone interaction F (15,80) = 9.162 and p<0.001, followed by Bonferroni posttests). Round 2–3 uM and −10 uM clones on average grow 54±8 and 50±16% slower compared to wild-type Dd2 clones, respectively. B. Growth of C53-1 (left plot, triangle) and C71-1a (right plot, diamond) during DSM1 removal experiments. Parasites were cultured in the presence (closed shape, solid line) or absence (open shape, dotted line) of DSM1 for 45 days before growth was measured as in (A) for an additional 14 days and plotted as Mean Normalized Parasitemia (normalization was performed against the maximum growth of the respective –DSM1 clone). Significance could not be determined because only a single value was measured for each time point. While there is no difference in growth between C53-1 ± DSM1, C710-1a (and 2b, data not shown) regains 56% of its growth rate. Growth of Dd2 (grey line) was included for comparison. (TIF) [file ppat.1003375.s007.tif]

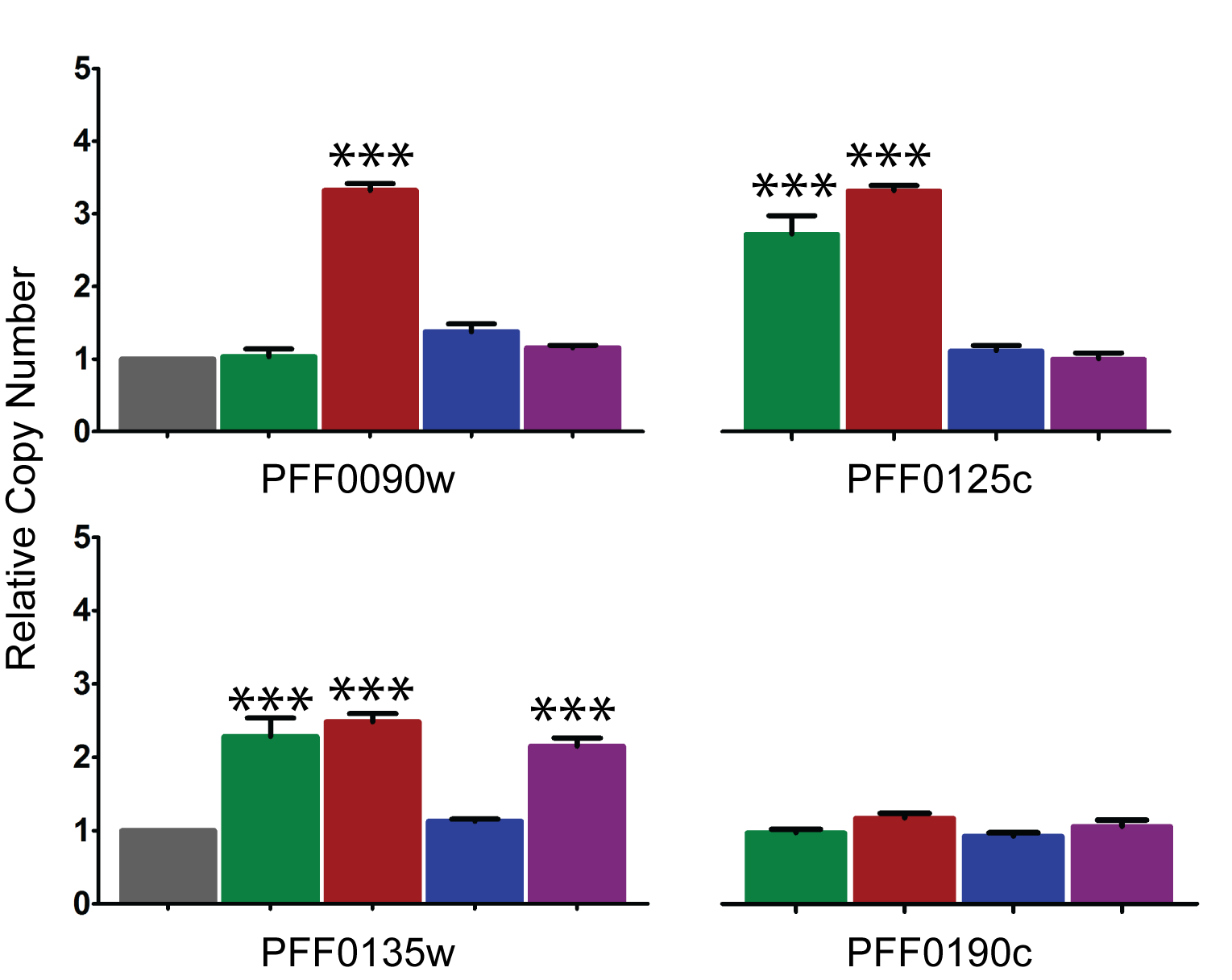

Supplement: Figure S8 — qPCR analysis of copy number of various genes across the amplified region of chromosome 6 (italicized genes in Table S7, primers in Table S12). C, green; D, red; E, blue; F, magenta. All values are relative to Dd2 (grey), normalized against seryl t-RNA synthetase copy number (data normalized to the 18 s ribosomal RNA gene displayed similar results), and determined from multiple experiments. Error bars depict standard error. Significance was determined against Dd2 (***, p value<0.0005). (TIF) [file ppat.1003375.s008.tif]

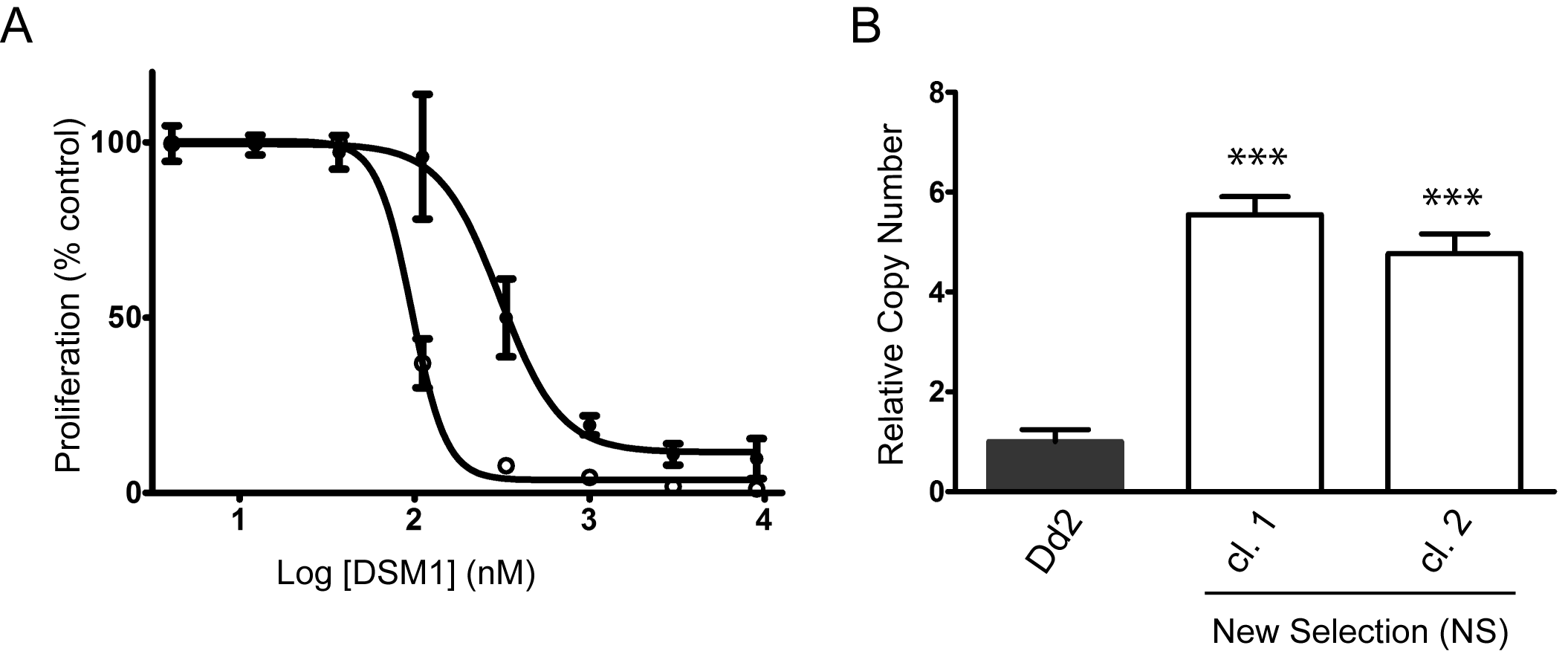

Supplement: Figure S9 — Characteristics of newly selected (NS) DSM1 resistant clones. A. EC50 plots comparing Dd2 (open circle, EC50 value: 0.1±0.01 µM) to uncloned parasites selected with 0.3 µM DSM1 (closed circle, EC50 value: 0.3±0.03 µM). Parasite proliferation was measured in triplicate using the hypoxanthine uptake assay and expressed as a percentage of total radioactivity count from the DMSO control. Error bars depict standard error. B. qPCR analysis of DHODH copy number in two NS clones (clone 1 mean 5.6±0.4, clone 2 mean 4.8±0.4). Significance was determined relative to Dd2 (***, p value<0.0005). (TIF) [file ppat.1003375.s009.tif]
