# Supplementary material for: Asexual Populations of the Human Malaria Parasite, Plasmodium falciparum, Use a Two-Step Genomic Strategy to Acquire Accurate, Beneficial DNA Amplifications
Source: PLoS Pathog. 2013 May 23;9(5):e1003375. doi: 10.1371/journal.ppat.1003375 (PMC3662640; doi:10.1371/journal.ppat.1003375)
Supplement: Table S1 — Summary of initial DSM1 challenge results. Dd2 (DSM1 EC50 of 0.2 µM) and HB3 (DSM1 EC50 of 0.06 µM) infected erythrocytes were challenged with various concentrations of DSM1 to determine the highest level of achievable resistance. Population sizes of 102–106 Dd2 or Hb3 parasites were also tested but not able to develop resistance to 0.1 µM or higher concentrations of DSM1 (unpublished data). (DOC) [file ppat.1003375.s010.doc]

|  | | Flasks Positive/Flasks Setup  (Days to observe parasites ±SD) | |
| --- | --- | --- | --- |
| **[DSM1] (µM)** | **Initial Population** | **Dd2** | **Hb3** |
| No Drug | 10^1^ | 3/3 (13±2) | 3/3 (28±0) |
| 0.1 | 10^1^ | 3/3 (39±0) | 0/3 |
| 0.1 | 10^7^ | 3/3 (39±0) | 0/3 |
| 0.3 | 10^1^ | 0/3 | 0/3 |
| 0.3 | 10^7^ | 3/3 (48±7) | 0/3 |
| 1.0 | 10^1^ | 0/3 | 0/3 |
| 1.0 | 10^7^ | 0/3 | 0/3 |
| 3.3 | 10^1^ | 0/3 | 0/3 |
| 3.3 | 10^7^ | 0/3 | 0/3 |
| 10 | 10^1^ | 0/3 | 0/3 |
| 10 | 10^7^ | 0/3 | 0/3 |
