# Supplementary material for: Asexual Populations of the Human Malaria Parasite, Plasmodium falciparum, Use a Two-Step Genomic Strategy to Acquire Accurate, Beneficial DNA Amplifications
Source: PLoS Pathog. 2013 May 23;9(5):e1003375. doi: 10.1371/journal.ppat.1003375 (PMC3662640; doi:10.1371/journal.ppat.1003375)
Supplement: Table S2 — Round 1 selections. 107 Dd2 parasites were plated over 24 wells (4 replicates) and challenged with 0.3 µM DSM1 (Note: populations of <107 parasites were not able to survive treatment with this DSM1 concentration). In total, 8 wells were positive for resistant parasites (round 1 clones). Four of these wells were randomly selected for DSM1 EC50 determination and sub-cloning. One sub-clone of each round 1 clone was selected for further analysis. Nd, EC50 not determined. (DOC) [file ppat.1003375.s011.doc]

| Parent clone/  Replicate Number | Round 1 Clone* | Sub-clone | Days to Detection | EC_50_ ± 95% CI (µM) |
| --- | --- | --- | --- | --- |
| Dd2^sensitive^ | -- | | 13 | 0.2±0.0 |
| 1 | B3** |  | 93 | -- |
| 2 | C12 |  | 85 | 1.0±0.1 |
|  |  | C12sC8 (C) | - | 1.1±0.1 |
|  | D9 |  | 36 | 0.9±0.2 |
|  |  | D9sD5 (D) | - | 0.9±0.2 |
|  | E1 |  | 52 | Nd |
|  | E4 |  | 66 | Nd |
| 3 | E10 |  | 17 | 1.2±0.1 |
|  |  | E10sD6 (E) | - | 0.9±0.2 |
|  | F2 |  | 22 | Nd |
|  | F4 |  | 88 | 1.0±0.1 |
|  |  | F4sH12 (F) | - | 0.9±0.1 |
| 4 | none |  | -- | -- |

*The clone name is based on the coordinates of the well in which it was isolated from the selection plate.

**This clone did not grow in 10 ml flask in presence of 0.3 µM DSM1.
