# Supplementary material for: Asexual Populations of the Human Malaria Parasite, Plasmodium falciparum, Use a Two-Step Genomic Strategy to Acquire Accurate, Beneficial DNA Amplifications
Source: PLoS Pathog. 2013 May 23;9(5):e1003375. doi: 10.1371/journal.ppat.1003375 (PMC3662640; doi:10.1371/journal.ppat.1003375)
Supplement: Table S3 — Whole genome sequencing coverage rates of various regions of interest across the P. falciparum genome. Overall very deep coverage was achieved in all clones except C710-1b clone (designated “*”). Whole genome rates are considerably lower than those for the DHODH gene presumably due to the inclusion of intergenic regions in this data set where base composition may limit the unique alignment of many reads. Coverage rates within clone C and D amplicon boundaries are included to emphasize the very deep coverage of these areas and thus, our confidence in the lack of mutations across these regions. Nd, not determined. (DOC) [file ppat.1003375.s012.doc]

|  |  | Covered by Number of Reads: | | | | | |
| --- | --- | --- | --- | --- | --- | --- | --- |
| Region of Genome | Clone | >1 | >5 | >10 | >20 | >50 | >200 |
| Whole Genome | Dd2 | 88.3% | 86.2% | 84.7% | 81.6% | 77.1% | Nd |
|  | C | 88.6% | 87.5% | 86.9% | 85.9% | 83.2% | Nd |
|  | D73-1 | 88.0% | 86.4% | 85.3% | 83.5% | 77.6% | Nd |
|  | C710-1b* | 83.9% | 66.9% | 53.8% | 32.6% | 1.3% | Nd |
|  | C710-2a | 87.7% | 84.9% | 82.4% | 77.2% | 61.6% | Nd |
| DHODH Gene | Dd2 | 100.0% | 100.0% | 100.0% | 100.0% | 100.0% | 30.2% |
|  | C | 100.0% | 100.0% | 100.0% | 100.0% | 100.0% | 100.0% |
|  | D73-1 | 100.0% | 100.0% | 100.0% | 100.0% | 100.0% | 100.0% |
|  | C710-1b* | 100.0% | 100.0% | 100.0% | 100.0% | 100.0% | 81.0% |
|  | C710-2a | 100.0% | 100.0% | 100.0% | 100.0% | 100.0% | 100.0% |
| Clone C  Boundaries | Dd2 | 98.9% | 98.0% | 96.8% | 94.2% | 83.7% | 7.5% |
|  | C | 99.4% | 99.1% | 98.9% | 98.8% | 98.5% | 95.5% |
|  | C710-1b* | 98.9% | 97.6% | 95.8% | 92.2% | 79.2% | 33.5% |
|  | C710-2a | 99.4% | 98.9% | 98.8% | 98.6% | 97.4% | 89.1% |
| Clone D Boundaries | Dd2 | 95.1% | 93.7% | 91.9% | 89.1% | 78.3% | 6.6% |
|  | D73-1 | 95.9% | 95.3% | 95.1% | 94.7% | 94.0% | 90.3% |
