# Supplementary material for: Asexual Populations of the Human Malaria Parasite, Plasmodium falciparum, Use a Two-Step Genomic Strategy to Acquire Accurate, Beneficial DNA Amplifications
Source: PLoS Pathog. 2013 May 23;9(5):e1003375. doi: 10.1371/journal.ppat.1003375 (PMC3662640; doi:10.1371/journal.ppat.1003375)
Supplement: Table S4 — Summary of mutations in the DHODH amplicon. In order to find low frequency mutations in an amplified region, positional nucleotide frequencies were identified by comparing Illumina reads from resistant clones and Dd2 that cover the amplicons on chromosome 6. This result of this analysis across the DHODH gene is also summarized in Fig. S4. DHODH (PFF0160c) is the target of DSM1. (−) no SNPs detected. (DOC) [file ppat.1003375.s013.doc]

|  | Round 1 | Round 2 | | |
| --- | --- | --- | --- | --- |
| Exon | C | C710-1b | C710-2a | D73-1 |
| PFF0095c | - | - | - | SNP85172 (T->A) SNP85177 (A->T) SNP85188 (T->C) |
| PFF0100w | - | - | - | - |
| PFF0105w | - | - | - | - |
| PFF0110w | - | - | - | - |
| PFF0115c | - | - | - | - |
| PFF0120w | - | - | - | - |
| PFF0125c | - | - | - | - |
| PFF0130c | - | - | - | - |
| PFF0135w | - | - | - | - |
| PFF0140c | - | - | - | - |
| PFF0145w | - | - | - | - |
| PFF0150c | - | - | - | - |
| PFF0155w | - | - | - | - |
| PFF0160c | - | - | - | - |
| PFF0165c | - | - | - | - |
| PFF0170w | - | - | - | - |
| PFF0175c | - | - | - | - |
| PFF0180w | - | - | - | - |
