# Supplementary material for: Asexual Populations of the Human Malaria Parasite, Plasmodium falciparum, Use a Two-Step Genomic Strategy to Acquire Accurate, Beneficial DNA Amplifications
Source: PLoS Pathog. 2013 May 23;9(5):e1003375. doi: 10.1371/journal.ppat.1003375 (PMC3662640; doi:10.1371/journal.ppat.1003375)
Supplement: Table S5 — SNPs present in >90% of Illumina sequencing reads. The top 5 listed SNPs were validated by PCR and sequencing (Table 1) and shown to preexist in Dd2 (see Materials and Methods section of main paper). (DOC) [file ppat.1003375.s014.doc]

|  |  |  |  |  | Round 1 | Round 2 | | |
| --- | --- | --- | --- | --- | --- | --- | --- | --- |
| Chr. | Position | AA Change | ID* | Description ** | C clone | D73-1 | C710-1a | C710-2b |
| 5 | 214244 | Cys->Ser | PFE0245c | Membrane protein | + | + | + | + |
| 6 | 645035 | Asn->Asp | PFF0750w | Cdc-related protein kinase | + | + | + | + |
| 8 | 738807 | Tyr->Asn | MAL8P1_  82 | Vacuolar  sorting protein | + | + | + | + |
| 5 | 214184 | Cys->Ser | PFE0245c | Membrane protein | + | + | (+) | + |
| 14 | 721985 | Lys->Stop | PF14_0173 | cAMP binding protein | + | + | - | + |
| 12 | 151552 | Lys->Ile | PFL0130c | Conserved/  unknown function | - | - | + | - |
| 8 | 902680 | Lys->Asn | PF08_0048 | Snf2-related CBP activator | - | - | + | - |
| 12 | 865888 | Met->Arg | PFL1045w | Conserved/  unknown function | - | - | - | + |
| 14 | 500065 | Ser->Asn | PF14_0123 | Conserved/  unknown function | - | - | - | + |
| 6 | 404586 | Ser->Phe | PFF0470w | Conserved/  unknown function | - | - | - | + |

*PlasmoDB gene ID

**Basic gene description based on PlasmoDB functional assignments.

(+) does not qualify for all conditions but SNP is present in C710-1a (covered by only 2 reads (cutoff for filtering is 5 reads))
