# Supplementary material for: Asexual Populations of the Human Malaria Parasite, Plasmodium falciparum, Use a Two-Step Genomic Strategy to Acquire Accurate, Beneficial DNA Amplifications
Source: PLoS Pathog. 2013 May 23;9(5):e1003375. doi: 10.1371/journal.ppat.1003375 (PMC3662640; doi:10.1371/journal.ppat.1003375)
Supplement: Table S6 — Summary of chromosome 6 amplicon boundaries and DHODH copy numbers for key round 1 and 2 clones. Microarray probe positions were judged from mid-density microarray analysis as the first (Start) and last (Stop) probe that exhibited a log2 ratio >0.3 in the amplicon border region. Mean log2 ratios were calculated across the entire amplified region. Since exact DHODH copy numbers could not be estimated from amplicons exhibiting log2 ratio >0.8, qPCR was employed (mean of values for front DHODH primer set (Table S12) from multiple experiments). Clones from two DSM1 removal (DR) experiments in which CGH analysis was performed are listed underneath the clone in which they were derived. All CGH experiments are pair-wise comparisons against Dd2 genomic DNA (except DR clones are compared to the round 1 C clone). Nd, not determined. (DOC) [file ppat.1003375.s015.doc]

|  |  | Microarray Probe Position | |  |  | DHODH Copy Number | |
| --- | --- | --- | --- | --- | --- | --- | --- |
| Round | Clone | Start | Stop | True Genome Region* | Mean Log_2_ Ratio | CGH^$^ | qPCR (±SE) |
| 1 | C | 129409% | 202401 | 79409-152456 | 0.8 | 3 | 4.0 ± 0.4 |
| 2 | C53-1 | 129209 | 202401 | 79209-152456 | 2.4 | >4 | 8.3 ± 0.5 |
|  | DR clone 4 | *129409^#^* | *202401* | *79409-152456* | - | - | 2.7 ± 0.3 |
|  | C73-1 | 129209 | 202401 | 79209-152456 | 2.5 | >4 | Nd |
|  | C710-1a | 129209 | 202401 | 79209-152456 | 2.5 | >4 | 10.0 ± 1.7 |
|  | DR clone 3 | 129209 | 202401 | 79209-152456 | - | - | 4.8 ± 0.4 |
|  | C710-1b | Nd | Nd | Nd | Nd | Nd | 12.2 ± 0.7 |
|  | C710-2b | 129209 | 202401 | 79209-152456 | 2.3 | >4 | 11.5 ± 0.5 |
| 1 | D | 114617 | 208017 | 64619-158072 | 0.9 | 4 | 3.5 ± 0.3 |
| 2 | D53-1 | 114617 | 208145^&^ | 64619-158204 | 2.0 | >4 | Nd |
|  | D53-2 | Nd | Nd | Nd | Nd | Nd | 8.6 ± 0.5 |
|  | D73-1 | 114617 | 208017 | 64619-158074 | 1.5 | >4 | 12.3 ± 1.2 |
|  | D73-2 | 114617 | 208017 | 64619-158074 | 2.2 | >4 | Nd |

*The genome region is different from probe position due to a misalignment of microarray probes by ~50kb in this region. This is likely due to the status of the alignment of the *P. falciparum* genome project at the time of microarray design.

^$^Based on NimbleGen log_2_ ratio scale: 0.25 to 0.5= 1 additional unit, 0.5 to 0.8= 2 additional units, >0.8= 3+ additional units.

%Probe 129409 is 2 probes away from 129209 on the mid-density microarray and does not likely represent a difference in the location of the amplicon junction.

^#^An approximation based on CGH comparison to round 1 clone C (in italics). Mean log_2_ ratio was not included because comparisons were made against the round 1 C clone instead of Dd2.

^&^Probe 208145 is 1 probe away from 208017 and does not likely represent a difference in the location of the junction.
