# Supplementary material for: Asexual Populations of the Human Malaria Parasite, Plasmodium falciparum, Use a Two-Step Genomic Strategy to Acquire Accurate, Beneficial DNA Amplifications
Source: PLoS Pathog. 2013 May 23;9(5):e1003375. doi: 10.1371/journal.ppat.1003375 (PMC3662640; doi:10.1371/journal.ppat.1003375)
Supplement: Table S7 — Description of genes contained within the largest DHODH amplicon. The DHODH target of DSM1 is bolded. Other genes selected for qPCR analysis (see Table S12) are italicized. The smallest amplicon (from clone E) encompasses gene numbers 15 to 23. (DOC) [file ppat.1003375.s016.doc]

| Gene Number* | ID** | Description*** |
| --- | --- | --- |
| 1^#^ | PFF0070w | PfEMP1 pseudogene |
| 2 | PFF0075c | PHISTb exported protein |
| 3 | PFF0080c | TRAP-like protein |
| 4 | PFF0085w | PHISTa exported protein |
| *5* | *PFF0090w* | *Conserved/unknown function* |
| 6 | PFF0095c | Conserved/unknown function |
| 7 | PFF0100w | ATP-dependent RNA helicase |
| 8 | PFF0105w | MYND finger protein |
| 9 | PFF0110w | Liver merozoite formation protein |
| 10 | PFF0115c | Elongation factor G |
| 11 | PFF0120w | Geranylgeranyl transferase |
| *12* | *PFF0125c* | *Conserved/unknown function* |
| 13 | PFF0130c | Conserved/unknown function |
| *14* | *PFF0135w* | *JmjC domain containing protein* |
| 15 | PFF0140c | Conserved/unknown function |
| 16 | PFF0145w | Conserved/unknown function |
| 17 | PFF0150c | Conserved/unknown function |
| 18 | PFF0155w | Mitochondrial chaperone BCS-1 |
| ***19*** | ***PFF0160c*** | ***DHODH*** |
| 20 | PFF0165c | Conserved/unknown function |
| 21 | PFF0170w | Cation/H+ antiporter (PfCHA) |
| 22 | PFF0175c | Conserved/unknown function |
| 23 | PFF0180w | Phenylalanyl-tRNA synthetase subunit |
| 24 | PFF0185c | Conserved/unknown function |
| *25^#^* | *PFF0190c* | *Conserved/unknown function* |

*****From Fig. 2B and C.

**PlasmoDB gene ID

***Basic gene description based on PlasmoDB functional assignments.

^#^Not included in longest DHODH amplicon (D).
