# Supplementary material for: Asexual Populations of the Human Malaria Parasite, Plasmodium falciparum, Use a Two-Step Genomic Strategy to Acquire Accurate, Beneficial DNA Amplifications
Source: PLoS Pathog. 2013 May 23;9(5):e1003375. doi: 10.1371/journal.ppat.1003375 (PMC3662640; doi:10.1371/journal.ppat.1003375)
Supplement: Table S8 — Round 2 selections. Populations of parasites were challenged with 1–10 µM DSM1 and scored for positive growth over 48 (for an initial population of 101) or 96 days (all other conditions). Resistant parasites from parental clone C and D were sub-cloned for further analysis (*) but those from clones E and F were not followed further. Nd, not determined. (DOC) [file ppat.1003375.s017.doc]

| Parent Clone/  Round 1 Clone | Initial Population | Wells Positive/Wells Setup  (Days to observed parasites) | | |
| --- | --- | --- | --- | --- |
|  |  | 1 µM | 3.3 µM | 10 µM |
| Dd2^sensitive^ | 10^1^ | 0/48 | 0/48 | Nd |
|  | 10^5^ | Nd | Nd | Nd |
|  | 10^7^ | 0/96 | 0/96 | Nd |
| C | 10^1^ | 0/48 | 0/48 | 0/48 |
|  | 10^5^ | Nd | 16/72 (24)* | Nd |
|  | 10^7^ | 72/72 (9) | 72/72 (19)* | 7/72 (25)* |
| D | 10^1^ | 0/48 | 0/48 | 0/48 |
|  | 10^5^ | Nd | 1/96* | Nd |
|  | 10^7^ | 96/96 (7) | 39/96 (18)* | 0/96 |
| E | 10^1^ | 0/48 | 0/48 | 0/48 |
|  | 10^5^ | Nd | 26/96 (23) | Nd |
|  | 10^7^ | 96/96 (7) | 96/96 (17) | 10/96 (26) |
| F | 10^1^ | 0/48 | 0/48 | 0/48 |
|  | 10^5^ | Nd | 24/96 (17) | Nd |
|  | 10^7^ | 96/96 (9) | 78/96 (22) | 25/96 (25) |
