# Supplementary material for: Asexual Populations of the Human Malaria Parasite, Plasmodium falciparum, Use a Two-Step Genomic Strategy to Acquire Accurate, Beneficial DNA Amplifications
Source: PLoS Pathog. 2013 May 23;9(5):e1003375. doi: 10.1371/journal.ppat.1003375 (PMC3662640; doi:10.1371/journal.ppat.1003375)
Supplement: Table S9 — Summary of EC50 values for round 2 clones. Values were determined using a higher range of DSM1 (0.09–200 µM) than was used for round 1 clones. These concentrations approach saturation for this compound, which may explain the increase in Dd2 EC50 (compared to value in Table S2) and high variability observed in these experiments. Fold increase was calculated against Dd2 from this table (tested at the high range of DSM1). Results from drug removal (DR) experiments are listed underneath the clone in which they were derived from (−1: 1 month without DSM1). Nd, could not be determined. (DOC) [file ppat.1003375.s018.doc]

| **Clone** | **Exp. No.** | **EC_50_ (**µ**M)** | **95% CI** | **Mean EC_50_ (**µ**M)** | **~Fold**  **Increase** |
| --- | --- | --- | --- | --- | --- |
| Dd2 | 1 | 0.3 | ±0.2 | 0.5 | **-** |
|  | 2 | 0.6 | ±0.2 |  |  |
|  | 3 | 0.3 | ±0.01 |  |  |
|  | 4 | 0.6 | ±0.02 |  |  |
| C53-1 | 1 | 9.5 | ±2.7 | 7.2 | **15** |
|  | 2 | 4.9 | ±1.2 |  |  |
|  | DR-1 | 1.5 | ±0.3 | - | 3 |
|  | DR-2 | 1.3 | ±0.8 |  | 2 |
|  | DR-3 | 1.7 | ±0.5 |  | 3 |
| C710-1a | 1 | 29 | ±12 | 62 | **130** |
|  | 2 | 66 | ±22 |  |  |
|  | 3 | 95 | ±11 |  |  |
|  | 4 | 59 | ±13 |  |  |
|  | DR-1 | 34 | ±12 | - | 60 |
|  | DR-2 | 3.2 | ±4.2 |  | 5 |
|  | DR-3 | 1.0 | ±0.3 |  | 2 |
| C710-1b | 1 | 48 | ±21 | 85 | **180** |
|  | 2 | 122 | ±93 |  |  |
| C710-2a | 1 | 65 | Nd | 56 | **120** |
|  | 2 | 46 | ±12 |  |  |
| C710-2b | 1 | 59 | ±20 | 53 | **115** |
|  | 2 | 52 | ±10 |  |  |
|  | 3 | 45 | ±14 |  |  |
|  | 4 | 59 | ±20 |  |  |
|  | DR-1 | 25 | ±10 | - | 40 |
|  | DR-2 | 1.9 | ±1.2 |  | 3 |
|  | DR-3 | 1.3 | ±2.3 |  | 2 |
| D53-1 | 1 | 9.1 | ±1.9 | 36 | **75** |
|  | 2 | 62 | ±11 |  |  |
| D53-2 | 1 | 65 | Nd | 65 | **140** |
| D73-1 | 1 | 49 | ±6.8 | 49 | **100** |
