# Supplementary material for: Asexual Populations of the Human Malaria Parasite, Plasmodium falciparum, Use a Two-Step Genomic Strategy to Acquire Accurate, Beneficial DNA Amplifications
Source: PLoS Pathog. 2013 May 23;9(5):e1003375. doi: 10.1371/journal.ppat.1003375 (PMC3662640; doi:10.1371/journal.ppat.1003375)
Supplement: Table S10 — EC50 values for DSM1 resistant round 1 clones D and E after 8 months of continuous culture with or without (*) 0.3 µM DSM1 pressure. Fold-resistance values are included in parentheses for ease of comparison. (DOC) [file ppat.1003375.s019.doc]

|  | DSM1 EC_50_ (µM) ± 95%CI (Fold-resistance) | | | | | | | | | |
| --- | --- | --- | --- | --- | --- | --- | --- | --- | --- | --- |
| Round 1 Sub-clone | **0 months** | | 2 months | | 4 months | | 6 months | | 8 months | |
| D* | **-** |  | 0.5±0.1 | (2.5) | 0.5±0.1 | (2.5) | 0.6±0.1 | (3) | 0.5±0.1 | (2.5) |
| D | **0.9±0.2** | **(4.5)** | 0.6±0.0 | (3) | 1.1±0.1 | (5.5) | 1.0±0.1 | (5) | 0.9±0.1 | (4.5) |
| E* | **-** |  | 0.6±0.1 | (3) | 0.7±0.2 | (3.5) | 0.5±0.1 | (2.5) | 0.5±0.1 | (2.5) |
| E | **0.9±0.2** | **(4.5)** | 1.0±0.1 | (5) | 1.5±0.3 | (7.5) | 0.9±0.1 | (4.5) | 1.3±0.3 | (6.5) |
