# Supplementary material for: Asexual Populations of the Human Malaria Parasite, Plasmodium falciparum, Use a Two-Step Genomic Strategy to Acquire Accurate, Beneficial DNA Amplifications
Source: PLoS Pathog. 2013 May 23;9(5):e1003375. doi: 10.1371/journal.ppat.1003375 (PMC3662640; doi:10.1371/journal.ppat.1003375)
Supplement: Table S11 — Additional antimalarial EC50 determination (±95% CI) for DSM1 resistant clones. Proguanil and 1843U89 target the P. falciparum dihydrofolate reductase enzyme and thymidylate synthase, respectively [72]. 5-Fluoroorotate inhibits pyrimidine biosynthesis in P. falciparum [73], [74]. Artemisinin is currently used clinically and its target remains unidentified (reviewed in [75]). Assay type A is a flow cytometry-based method that involves measurement of parasitemia using SYBR green and assay type B depends on the uptake of radiolabeled hypoxanthine (see Materials and Methods and Text S1). (−), experiment not performed. Nd, could not be determined. (DOC) [file ppat.1003375.s020.doc]

| Parasite Clone/  Antimalarial | Proguanil EC_50_ (µM) | 5-Fluoroorotate EC_50_ (nM) | | 1843U89  EC_50_ (nM) | Artemisinin  EC_50_ (nM) | |
| --- | --- | --- | --- | --- | --- | --- |
| Assay Type | A | A | B | B | A | B |
| Dd2 | 12.4 (±0.9) | 4.5 (±0.2) | 7.9 (±0.3) | 290 (Nd) | 12.5 (Nd) | 11.5 (±1.4) |
| C | 14.8 (±3.6) | 5 (±0.4) | - | - | 8.3 (±0.8) | - |
| D | 14.8 (±3.4) | 5.2 (±0.6) | - | - | 7.4 (±1) | - |
| C53-1 | 10.3 (±4.4) | 3.6 (Nd) | - | - | 12.5 (Nd) | - |
| D73-1 | - | - | - | - | 4.7 (±0.5) | - |
| D73-2 | 7 (±2.1) | - | 8.3 (Nd) | 244 (±24.1) | - | 4.4 (±0.7) |
| C710-1a | 10.3 (±2.7) | 3.3 (Nd) | - | - | 5 (Nd) | - |
| C710-2a | 17.6 (±3.9) | 5.1 (±0.5) | - | - | 5.4 (±0.4) | - |
