# Supplementary material for: Asexual Populations of the Human Malaria Parasite, Plasmodium falciparum, Use a Two-Step Genomic Strategy to Acquire Accurate, Beneficial DNA Amplifications
Source: PLoS Pathog. 2013 May 23;9(5):e1003375. doi: 10.1371/journal.ppat.1003375 (PMC3662640; doi:10.1371/journal.ppat.1003375)
Supplement: Table S12 — Summary of primers by experiment. (DOC) [file ppat.1003375.s021.doc]

| **Exp.** | **Details** | | | **Primer Sequence** | **Product size** |
| --- | --- | --- | --- | --- | --- |
| qPCR | Gene ID | | Function |  |  |
|  | PFF0090w | | Unknown | F-CCAAAATGTCAAAACACTATG  R-CTGCATTGGCTGAAGCATAAACAG | 158bp |
|  | PFF0125c | | Unknown | F-CGTCCATGAATGTGAAGAGTGG  R-GGATAAGTAGATACAACACTAC | 237bp |
|  | PFF0135w | | Unknown | F-CAGCCAGGACATACGAAGAGG  R-GCATTGCCCTATCTTATCTTG | 163bp |
|  | PFF0160c | | DHODH Front  DHODH Rear | F-TCCATTCGGTGTTGCTGCAGGATTTGAT  R-TCTGTAACTTTGTCACAACCCATATTA  F-GTGTTAGCGGAGCAAAACTAAAAG  R-ATAATTGACAAACTGAAGCACCTG | 206bp  158bp |
|  | PFF0190c | | Unknown | F-GACGATATTCAGAATGATGTTCAG  R- TTTACGATCTTCTTTAACACACC | 175bp |
|  | PF07_0073 | | Seryl t-RNA Synthetase | F-GGAACAATTCTGTATTGCTTTACC  R- AAGCTGCGTTGTTTAAAGCTC | 142bp |
|  | PFL1155w | | GTP cyclohydrolase I | F-AAATATGAGGGGAGTTAAAGAGCA  R- TTTAAATTTTCCACAGAAGAGTCA | 120bp |
|  | MAL13P1.435 | | 18s Ribosomal RNA | F-ACAATTCATCATATCTTTCAATCGGTA  R- GCTGACTACGTCCCTGCCC | 69bp |
| Junction PCR | C junction | | | *1-CGGATGCTCATCACAAAAGA**  2-TCAAAGGAGAGTCCCAAAGG | ~1600bp |
|  | D junction | | | 1-CTGCTGATGGCTAAATTCTCA  2-GACCGTGTGTTGAATAGTTTCTTT | ~250bp |
|  | E junction | | | 1-CAGTGAAATCTGGAAAGACGAG  2-TGGATAAACAGGTTGAAAAAGAG | ~400bp |
|  | F junction | | | 1-CGGATGCTCATCACAAAAGA**  2-TGTTAATTCCGGGGTTACCTT | ~350bp |
| DHODH Seq. | DHODH-F | | | CATTTAAGCCCCAAAACATTTTTAC | N.A. |
|  | DHODH-R | | | GTGATAGATAGCTCCAGTCGATTTC | N.A. |
|  | Seq1 | | | TCATCATATGTATCTGTACCTTTTAAGATT | N.A. |
|  | Seq2 | | | AGCTCCCCTAATACACCTGGGTT | N.A. |
|  | Seq3 | | | TGCAAAACCACGTATTTTTAGAGAC | N.A. |
|  | Seq4 | | | TATATATATATTTTTTTTTTTTTGCGC | N.A. |
|  | Seq5 | | | GCCCTTGGTTTTTGTTAAGTTAGCTCC | N.A. |
|  | Seq6 | | | TCTGTAACTTTGTCACAACCCATATTA | N.A. |
| SNP Validation | PFE0245c | Position 214184*** | | F-TCCTCTTCTTTTCTACATGCTACATC  R- TAATAAGAATAGGTGGAGACCTTTTTG | ~1300bp |
|  | PFE0245c | Position 214244*** | | F-TCCTCTTCTTTTCTACATGCTACATC  R- TAATAAGAATAGGTGGAGACCTTTTTG | ~1300bp |
|  | PFF0750w | Position 645035 | | F-GCATCATCATAAAAGTCATGCAA  R- AATGCATGCAGCTGACCATA | ~400bp |
|  | MAL8P1.82 | Position 738807 | | F-CGTTCGAAATTAATTCCTTCCA  R- GAAAAGCCTCCAAAAGGGATA | ~1000bp |
|  | PF14_0173 | Position 721985 | | F-AAGGCCAAAATTTGTCATCTG  R- CAGTATCGATTATTGCCACTGC | ~700bp |

*Primer number refers to the position of the primer on Fig. S5A. All primers numbered 1 are the forward direction and primers numbered 2 are the reverse direction for the PCR reaction.

**The same primer 1 was used because exact 3’ junction was predicted by microarray data for C and F clones.

***These SNPs were amplified using the same primers and sequenced in the same reaction since they are only 60 bp apart.

N.A. not applicable
