# Supplementary material for: Asexual Populations of the Human Malaria Parasite, Plasmodium falciparum, Use a Two-Step Genomic Strategy to Acquire Accurate, Beneficial DNA Amplifications
Source: PLoS Pathog. 2013 May 23;9(5):e1003375. doi: 10.1371/journal.ppat.1003375 (PMC3662640; doi:10.1371/journal.ppat.1003375)
Supplement: Table S13 — Copy number assessment of the chromosome 5 and 12 amplicons in DSM1 resistant clones using microarray and qPCR. (DOC) [file ppat.1003375.s022.doc]

|  |  | Chrom. 5 amplicon^*^ | | Chrom. 12 amplicon^*^ | | |
| --- | --- | --- | --- | --- | --- | --- |
| Round | Clone | Mean log_2_ ratio^#^ | Copy number^$^ | Mean log_2_ ratio | Copy number | qPCR% |
| - | Dd2 | - | 3^&^ | - | 2^&^ | 1.6 |
| 1 | C | 0.2 | 3 | 0.5 | 4 | 4.4 |
| 2 | C53-1 | 0.0 | 3 | 0.3 | 3 | 1.5@ |
|  | DR clone 4 | *0.0* | *3* | *0.5* | *4* | *Nd* |
|  | C73-1 | 0.2 | 3 | 0.6 | 4 | 3.3 |
|  | C710-1a | 0.1 | 3 | 1.2 | 5 | 4.2 |
|  | DR clone 3 | *0.0* | *3* | *-0.7* | *1* | *Nd* |
|  | C710-2b | 0.1 | 3 | 0.5 | 3 | 3.8 |
| 1 | D | -0.2 | 3 | -0.2 | 2 | 1.8 |
| 2 | D53-1 | -0.4 | 2 | 0.7 | 4 | 4.3 |
|  | D73-1 | -0.3 | 2 | 0.0 | 2 | 2.1 |
|  | D73-2 | -0.4 | 2 | 0.0 | 2 | 1.8 |

^*^Boundaries identified from WGS studies: Chromosome 5 (888060-970427) and chromosome 12 (971307-976534)

^#^All clones were compared to Dd2 using CGH (except DR clones (*italics*) were compared to the parental C clone).

^$^Approximate copy number was calculated based on NimbleGen log_2_ ratio scale: 0.25 to 0.5= 1 additional unit, 0.5 to 0.8= 2 additional units, >0.8= 3+ additional units on top of the number of copies already in Dd2.

%Copy number of this region was measured by qPCR using primers against GTP cyclohydrolase (PFL1155w, Table S9). Values are relative to parasite clone FCR3 which has a single copy of this region of chromosome 12 [56].

^&^Levels of the chromosome 5 and 12 amplicons in Dd2 were estimated from WGS studies and qPCR of PFL1155w respectively.

@This clone may have lost copies following subsequent rounds of culture.
