# Supplementary material for: Asexual Populations of the Human Malaria Parasite, Plasmodium falciparum, Use a Two-Step Genomic Strategy to Acquire Accurate, Beneficial DNA Amplifications
Source: PLoS Pathog. 2013 May 23;9(5):e1003375. doi: 10.1371/journal.ppat.1003375 (PMC3662640; doi:10.1371/journal.ppat.1003375)
Supplement: Text S1 — Supplementary Materials and Methods. This document contains the following sections: Expression Analysis, Determination of DHODH Protein Levels, Targeted DHODH Sequencing, EC50 Determination by SYBR Green Assay, PCR and Sequencing of the DHODH Amplicon Junction, Growth Assessment, Quantitative PCR of other genes contained within the DHODH amplicon, Abbreviations, and Supplementary References. (DOCX) [file ppat.1003375.s023.docx]

**Text S1 (Supplementary Information):**

Supplementary Materials and Methods

Abbreviations

Supplementary References

**Supplementary Materials and Methods**

*Expression Analysis*. Parasites were synchronized with 5% sorbitol for two successive cycles and then total RNA was isolated from cultures containing predominantly trophozoite stage using the RNAqueous kit (Ambion). cDNA was synthesized in the presence of aa-dUTP and then coupled to either Cy3 (DSM1-sensitive Dd2) or Cy5 (DSM1-resistant clones) [1,2]. Hybridizations of each pair to spotted DNA microarrays were performed at 62°C for 16-18h, after which, microarrays were washed, dried, scanned, and analyzed as described for CGH.

*Determination of DHODH Protein Levels. P. falciparum* DHODH was expressed and purified as the N-terminally truncated protein (to remove the membrane spanning domain) as previously described [3,4] and protein (20 mgs) was supplied to Affinity BioReagents (Golden, CO, USA) in 1 ml buffer (100 mM Hepes pH 8.0, 300 mM NaCl, 15% glycerol, 15% triton-reduced) for the generation of rabbit antibodies. Round 1 DSM1 resistant parasite were harvested and run on an SDS-PAGE gel for Western blot analysis. Rabbit anti-recombinant DHODH (1:7000) and goat anti-rabbit horseradish peroxidase (Jackson Laboratories, 1:10,000) were used as primary and secondary antibodies, respectively.

*Targeted DHODH Sequencing*. Genomic DNA was purified from each DSM1 resistant clone and the sensitive Dd2 clone using the method described in the paper. The DHODH gene was PCR amplified using DHODH-F and DHODH-R (Table S12). The optimized protocol, giving a single amplified product, was found to be 96^o^C for 3 min, 30 rounds of 96^o^C for 45 sec, 53^o^C for 1.5 min, and 68^o^C for 2 min, with a final extension of 68^o^C for 10 min. Amplified product was PCR-purified (Qiagen) and dideoxy-sequenced (Eurofins MWG Operon) using 6 additional primers (listed in Table S12) designed to walk across the entire gene with significant overlap. Consensus sequences were compiled for each clone and compared using Geneious Pro 5.5.6 (Fig. S3).

*EC_50_ Determination by SYBR Green Assay.* A parasite solution at 0.5% parasitemia (0.5% hematocrit) from the clone of interest was plated into a 96-well culture plate. An appropriate range of antimalarial concentrations were then added to the parasites (final 0.5% DMSO). Each concentration was performed in triplicate and included solvent-only and uninfected red blood cell controls. After incubating for ~72 hours, the majority of media was removed from each well (leaving red blood cells settled at the bottom). An equal volume of 2X SYBR green (diluted in PBS, Sigma Aldrich) was then added to each well and incubated at room temperature for 20 min before adding cold PBS up to 200µl. 96-well plates were stored at 4°C until parasitemia measurement was performed using the Accurri C6 flow cytometer with a CSampler robotic arm (BD Biosciences). 50,000 counts were collected from each well and gates to exclude debris and aggregates were set similar to [5]. Parasite proliferation in each test well was expressed as a percentage of the solvent control well. EC_50_ values were fit using the GraphPad PRISM software, according to the equation: Y = Bottom + (Top-Bottom) / (1+10^((LogEC50 - X) * HillSlope)^).

*PCR and Sequencing of the DHODH Amplicon Junction.* Genomic DNA was isolated from clones as described in the paper for microarrays using the DNeasy kit (Qiagen). Using unique primers sets for each round 1 clone (Table S12), we amplified the amplicon junction using the following protocol: 94°C 45 sec, 52-55°C 45 sec, 72°C 1 min. PCR products were cloned into the Topo-TA vector (Invitrogen) and transformed into bacteria. Single colonies were isolated (~4 per round 1 clone) and grown for minipreps (Qiagen) prior to di-deoxy sequencing using TOPO-TA-specific M13F/R primers.

*Growth Assessment.* Due to experimental variability, growth of DSM1 resistant clones were assessed for 2 to 4 parasite clones in 2 to 3 independent experiments and data were combined to determine an overall trend for each group of resistant parasites (i.e. round 1, round 2- 3 μM, and round 2- 10 μM). Percent parasitemia was recorded daily from thin smears, adjusted based on dilution of the culture, and normalized to the maximum growth of the Dd2 clone in each experiment. SEM was determined from the mean of multiple experiments and significance was determined by two-way ANOVA statistical test followed by a Bonferroni posttest. The relative growth rate was calculated by normalizing the average slope of the linear fit of percent parasitemia for each clone to the Dd2 values in each experiment. In the case of DSM1 removal experiments, parasitemia measurements began after 45 days of growth in the absence of DSM1 and normalization was performed against the maximum growth of the respective – DSM1 clone (*i.e*. growth of C53-1 +DSM1was normalized to growth of C53-1 –DSM1).

*Quantitative PCR of other genes contained within the DHODH amplicon*. The same qPCR protocol that was used to amplify DHODH was used for PFF0135c and PFF0190c (primer sequences are listed in Table S12). For PFF0090w and PFF0125c, an annealing temperature of 53^o^C was required. We performed melt curves, standard curves, and analysis as described in the main body of the paper.

**Abbreviations**

DHODH, dihydroorotate dehydrogenase; gDNA, genomic DNA; CGH, comparative genomic hybridization; WGS, whole genome sequencing; qPCR, quantitative PCR; SNP, single nucleotide polymorphism; NS, newly selected; Nd, not determined; DR, drug removal.

**Supplementary References**

1. Ganesan K, Jiang L, Rathod PK (2002) Stochastic versus stable transcriptional differences on Plasmodium falciparum DNA microarrays. International Journal for Parasitology 32: 1543-1550.

2. Ganesan K, Ponmee N, Jiang L, Fowble JW, White J, et al. (2008) A Genetically hard-wired metabolic transcriptome in *Plasmodium falciparum* fails to mount protective responses to lethal antifolates. PLoS Pathog 4: e1000214.

3. Phillips MA, Gujjar R, Malmquist NA, White J, El Mazouni F, et al. (2008) Triazolopyrimidine-based dihydroorotate dehydrogenase inhibitors with potent and selective activity against the malaria parasite Plasmodium falciparum. Journal of Medicinal Chemistry 51: 3649-3653.

4. Gujjar R, Marwaha A, El Mazouni F, White J, White KL, et al. (2009) Identification of a metabolically stable triazolopyrimidine-based dihydroorotate dehydrogenase inhibitor with antimalarial activity in mice. Journal of Medicinal Chemistry 52: 1864-1872.

5. Malleret B, Claser C, Ong AS, Suwanarusk R, Sriprawat K, et al. A rapid and robust tri-color flow cytometry assay for monitoring malaria parasite development. Sci Rep 1: 118.
